# Increased importance of cool‐water fish at high latitudes emerges from individual‐level responses to warming

**DOI:** 10.1002/ece3.10185

**Published:** 2023-06-06

**Authors:** Aslak Smalås, Raul Primicerio, Kimmo K. Kahilainen, Petr M. Terentyev, Nikolay A. Kashulin, Elena M. Zubova, Per‐Arne Amundsen

**Affiliations:** ^1^ Faculty of Bioscience, Fisheries and Economy, UiT The Arctic University of Norway Tromso Norway; ^2^ Scandinavian Nature Surveillance (Skandinavisk Naturovervåking) Åkerblå Group AS Tromsø Norway; ^3^ Lammi Biological Station University of Helsinki Helsinki Finland; ^4^ Institute of the Industrial Ecology Problems of the North (INEP) KSC RAS Apatity Russia

**Keywords:** age at maturation, climate change, growth, life history, perch, population dynamics, recruitment, thermal guilds

## Abstract

High latitude ecosystems are experiencing the most rapid warming on earth, expected to trigger a diverse array of ecological responses. Climate warming affects the ecophysiology of fish, and fish close to the cold end of their thermal distribution are expected to increase somatic growth from increased temperatures and a prolonged growth season, which in turn affects maturation schedules, reproduction, and survival, boosting population growth. Accordingly, fish species living in ecosystems close to their northern range edge should increase in relative abundance and importance, and possibly displace cold‐water adapted species. We aim to document whether and how population‐level effects of warming are mediated by individual‐level responses to increased temperatures, shift community structure, and composition in high latitude ecosystems. We studied 11 cool‐water adapted perch populations in communities dominated by cold‐water adapted species (whitefish, burbot, and charr) to investigate changes in the relative importance of the cool‐water perch during the last 30 years of rapid warming in high latitude lakes. In addition, we studied the individual‐level responses to warming to clarify the potential mechanisms underlying the population effects. Our long‐term series (1991–2020) reveal a marked increase in numerical importance of the cool‐water fish species, perch, in ten out of eleven populations, and in most fish communities perch is now dominant. Moreover, we show that climate warming affects population‐level processes via direct and indirect temperature effects on individuals. Specifically, the increase in abundance arises from increased recruitment, faster juvenile growth, and ensuing earlier maturation, all boosted by climate warming. The speed and magnitude of the response to warming in these high latitude fish communities strongly suggest that cold‐water fish will be displaced by fish adapted to warmer water. Consequently, management should focus on climate adaptation limiting future introductions and invasions of cool‐water fish and mitigating harvesting pressure on cold‐water fish.

## Graphical abstract

We studied 11 cool‐water adapted freshwater fish populations in communities dominated by cold‐water adapted species to investigate changes in the relative importance of cool‐water fish during the last 30 years of rapid warming in high latitude lakes. Our long‐term series (1991–2020) reveal a marked increase in numerical importance of the cool‐water fish species, perch, in ten out of eleven populations, and in most fish communities, the cool‐water species is now dominant. Moreover, we show that climate warming affects population‐level processes via direct and indirect temperature effects on individuals.

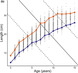

## 1 INTRODUCTION

High latitude regions are experiencing the most rapid warming on Earth, a trend projected to continue towards year 2100 (IPCC, 2013; Parmesan, 2006). Rapid warming strongly affects freshwater ecosystems (O'Reilly et al., 2015), leading to changes in species abundance, phenology, and distribution (Campana et al., 2020; Comte et al., 2013; Hickling et al., 2006). Temperature‐driven changes in physiological rates of ectotherms are expected to trigger a diverse array of ecological responses (Biro et al., 2007; Huss et al., 2019; Rezende & Bozinovic, 2019), with implications for ecosystem structure and function (Benateau et al., 2019). Fish are strongly influenced by ambient temperature, with species displaying distinctive thermal niches (Magnuson et al., 1979). Climate warming tends to favor fish populations currently experiencing the cold end of their species' thermal range, typically in proximity to the northern limits of species distribution (Campana et al., 2020; Ficke et al., 2007). As temperature increases, these populations are likely to outperform competing species of colder temperature guilds (Hayden et al., 2017; Hein et al., 2014). Evidence in support of, or against, these expectations is presently lacking due to a paucity of long‐term ecological studies of freshwater fish communities in the rapidly warming Arctic (Amundsen et al., 2019; Zubova et al., 2020).

The impact of increasing temperatures on fish populations is mediated by direct ecophysiological effects and indirect life‐history responses that ultimately affect survival and reproduction. In ectotherms, temperature limits biological rates, affecting for instance food intake and metabolism and their balance determining the net energy gain of an organism (Jobling, 2002). Growth rate will therefore depend on ambient temperature, with maximum growth being reached at an intermediate, optimum temperature within the thermal niche of a species (Gvoždík, 2018). The growth rate of individuals living at temperatures below their optimum might increase with climate warming, given sufficient food availability (Huss et al., 2019; Smalås et al., 2020). Higher juvenile growth rates lead to larger size at age and earlier maturation. Larger size at age might increase survival, especially during early life stages, because mortality in fish is largely size‐dependent (Hurst, 2007; Perez & Munch, 2010). Thus, faster growth increases the probability of reaching maturity, which is further enhanced by earlier maturation, overall resulting in higher transition rates to the adult, reproductive stage. Recruitment rates can be further enhanced by faster somatic growth as young adult females become larger, thereby producing larger clutches, thus climate warming is likely to boost population fecundity (Heibo et al., 2005). In addition, some critical life stages, in particular the egg and larvae, often have a narrower temperature range for survival and successful development than other life stages (Dahlke et al., 2020; Hokanson, 1977). Populations living close to the northern end of their distribution, might in colder years suffer from temperatures that are too low for successful development, especially in critical periods such as survival over the first winter, and should therefore benefit from climate warming (Dahlke et al., 2020; Hurst, 2007).

The effects of climate warming on high latitude lakes go beyond increasing water temperatures, and predicted changes in the aquatic environments, such as increased productivity, decreased dissolved oxygen levels, and altered seasonality, are likely to favor cool‐water species more than cold‐water fish (Ficke et al., 2007; Rolls et al., 2017). Increase in temperature and productivity will expectedly first favor percids, and later cyprinids, over salmonids (Hayden et al., 2017). These cool‐water species have been shown to redistribute northwards and to higher altitudes over the last few decades of rapid warming (Comte et al., 2013; Hayden et al., 2014; Rolls et al., 2017). Therefore, there is a compelling need to document changes but also to understand the mechanisms behind climate‐driven changes in high latitude fish communities in order to develop climate adaptation strategies that mitigate the possible eradication or displacement of cold‐water species in the Arctic. One cool‐water species moving northwards is the Eurasian perch (*Perca fluviatilis*), hereafter perch (Hayden et al., 2014), which has its northern range edge in subarctic regions of Eurasia around 70°N but with a wide temperature range for growth, between 5 and 33°C, and an optimum between 16 and 27°C (Hokanson, 1977; Karås, 1990) (more detailed information in Supplementary information).

Here, we address the effects of climate warming on perch populations at the northern end of the species distribution, using long‐term surveys of high latitude freshwater fish communities (68–70°N). As a cool‐water adapted species, we expect perch to benefit from increasing temperatures and a prolonged productive season, leading to increased abundance and importance relative to cold‐water adapted fish co‐inhabiting the sampled lakes. Several mechanisms underlie our expectation of an increase in the relative importance of perch following the recent rapid warming. Specifically, we focus on the life‐history and ecological implications of temperature‐induced increase in somatic growth rate, anticipating that higher growth rates (i) reduce the duration of critical life stages, (ii) increase size at age, and (iii) promote earlier maturation age; overall improving survival and increasing recruitment rates and total population fecundity (see Supplementary information, Figure S2 for schematic representation of mechanisms).

## 2 MATERIALS AND METHODS

### 2.1 Study area, fish sampling, and community‐level data

The study area is concentrated in northern Fennoscandia, with lakes situated in Norway, Finland, and Russia. All lakes are located north of the Arctic Circle, towards the northern end of the distribution of cool‐water fish species (>68°N) (See Supplementary information, Figure S3, for map of the study lakes and area). To assess the relative importance of Eurasian perch, we compiled population and community‐level data from 11 lakes sampled on multiple occasions over the last 30 years. The eleven study lakes were sampled between 2 and 26 times, with time series ranging from 8 to 32 years (Appendix Table S1). The total number of sampled fish across the 11 lakes was nearly 60,000, of which 12,000 were perch, and the littoral catches from this data were used to describe the development in proportion of perch in the 11 lakes (Appendix Table S1, the fish community of the individual lakes are described in the Supplementary information). Fish were collected using multimesh gill‐nets or gill‐net series in all three sampled habitats (i.e., littoral, pelagic, profundal) of the lakes, but only fish from the littoral zone were included in the analyses because the sampling in the other habitats was more scattered in both time and space (See supplementary information for details on the fish sampling procedure).

### 2.2 Individual‐level data

To assess changes in life‐history parameters in relation to water temperature in the perch over time, we used individual‐level data from the two lakes most frequently and intensively sampled (Lake Vaggatem and Lake Skrukkebukta). These two lakes were selected because of their long time series (~1991–2020) with mostly annual sampling, including daily data on water temperature, and detailed individual data collected from year 2003 to 2020. A total of 2960 individual perch from Lake Vaggatem (*n* = 38–608 per year) and Lake Skrukkebukta (*n* = 30–130) were sampled. The individual fish sampling procedure and summary information on individual‐ and population‐level data are described in the supplementary information Appendix S1 (see Tables S2 and S3).

### 2.3 Temperature

**FIGURE 1:**
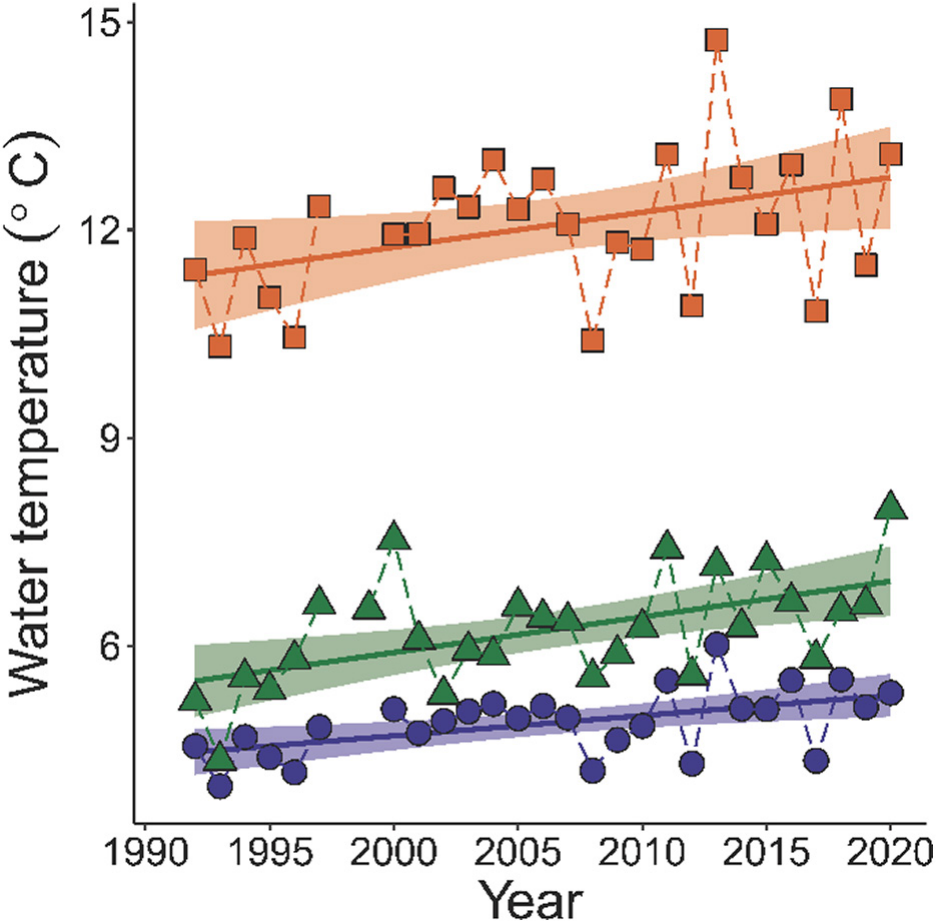
Water temperature in the Pasvik watercourse from year 1992 to 2020. Annual mean temperature (blue circles). Mean autumn temperature (Sep–Nov) (green triangles). Mean summer temperature (Jun–Aug) (orange squares). Temperature data are missing from January 1998–July 1999.

For the two main study systems, Lake Skrukkebukta and Lake Vaggatem, daily water temperature measurements were available from an automated datalogger at the Skogfoss hydropower plant situated 25 km upstream from Lake Skrukkebukta and 23 km downstream from Lake Vaggatem. We calculated annual mean water temperature, mean summer temperature (Jun–Jul–Aug), and mean autumn temperature (Sep–Oct–Nov) from the logger data. Mean annual water temperature has increased significantly by 0.3°C per decade (*p* < .01, *F* = 9.361_1,25_, adj‐*R* ^2^ = .243, Appendix Table S4), mean autumn temperature (September–November) has also increased significantly by 0.4°C per decade (*p* < .01, *F* = 11.26_1,26_, adj‐*R* ^2^ = .275, Appendix Table S5), and mean summer water temperature increased (June–August) by 0.4°C per decade (Figure 1) (*p* = .037, *F* = 4.87_1,25_, adj‐*R* ^2^ = .13, Appendix Table S6).

### 2.4 Statistical analysis

The proportion of perch in the fish community was calculated as the number of perch caught relative to the total number of fish caught in the littoral zone of the different lakes. To estimate how the proportion of perch in the catches changed over time we fitted a generalized linear mixed‐effect model (GLMM) with location (lakes) as a random effect and a binomial family handling proportional data. We used the glmer function from the lme4 package in R. Relative density of fish in terms of Catch‐Per‐Unit‐Effort (CPUE) was readily available for the two main study systems, Lake Skrukkebukta and Lake Vaggatem. The CPUE was calculated as the number of fish caught per 100 m^2^ gill‐net per night (or 12 h). To investigate the change in relative density over time, we estimated separate linear models for the two lakes, with log (CPUE+1) as the response and year as the predictor. To estimate the relationship between relative density and water temperature, we again used CPUE on a natural logarithmic scale with annual mean water temperature as a predictor in a linear regression model. We transformed the predictor to a weighted‐moving‐average over the last 2 years with double weighting given to the latest year, to better reflect any long‐term effects of temperature on relative density of perch. To investigate recruitment in the perch population, we used the relative proportion of 1‐year‐old individuals within the perch population as a measure of the number of individuals surviving the first winter, thus used as a proxy for recruitment of juveniles to the perch population. Juvenile recruitment is suggested to be dependent on water temperature (Dahlke et al., 2020) but also food availability (or density of competitors) and density of predators in their first growth season. Relative proportion of one‐year‐old individuals is calculated as the proportion of one‐year‐old perch relative to the total CPUE of the perch populations. We used generalized linear regression model (binomial distribution) with summer water temperature the preceding year to predict the relative proportion of one‐year‐old individuals in the perch populations. In addition, we used the CPUE of 1‐year‐olds directly to further investigate the recruitment of juveniles to the perch populations. We modeled how CPUE of one‐year‐old perch was related to summer water temperature the preceding year in a linear model with the response on a natural logarithmic scale. In the statistical analyses, we combined both lakes as the number of sampling points was too low to treat them separately.

### 2.5 Back‐calculated length‐at‐age and length increment

The back‐calculated length increment was estimated for a sub‐sample of perch from Lake Vaggatem and Lake Skrukkebukta. On the opercular bones of individual perch, we measured the width of annual growth increments as the distance between the opaque zones. We used these measurements in addition to the total opercular length and the body length of the individual perch to estimate length‐at‐age with the nonlinear body‐proportional hypothesis method. This method is commonly used for perch (Thoresson, 1996) and assumes that the deviation in body size from the expected body size given by the operculum size does not change through life (Thoresson, 1996),

$$L_{a}=L_{A}{\left(\frac{O_{a}}{O_{A}}\right)}^{\beta_{1}},$$

where $L_{a}$ is the back‐calculated length‐at‐age a, $O_{a}$ the measured operculum radius at age a, $O_{A}$ the observed operculum size at time of capture, and $L_{A}$ the observed fish length at time of capture. $\beta_{1}$ is the linear regression slope coefficient estimated from the log–log relationship between the body length and operculum length at capture. A total of 1646 perch were used in the back‐calculation procedure. We back‐calculated length increment (mm·year^−1^) for juvenile fish in the age group of 1–4 years with individual perch ranging from age 2 to 10 years, giving us length increment data from year 1995 to year 2018. We did not assess growth during the sampling year, because those estimates would be dependent on sampling time within the year, which was not exactly the same every year. A comparison between back‐calculated length at final winter before capture and length at capture revealed a good fit of the back‐calculation model (*p* < .001, *F* = 2501 on 1 and 1644 df, adj‐*R* ^2^ = .94) (Appendix Figure S6 and Table S9). Back‐calculated length increment (mm·year^−1^) for 1‐, 2‐, 3‐ and 4‐year‐old perch were related to summer water temperature and relative density in a linear mixed‐effect model (LME) with sampling year and age at capture as random effects, using the nlme package in R. In addition, we estimated cohort mean (year class) length increment from age 1 to 4, which was related to mean summer water temperature (°C) and mean relative density (CPUE) for the same time period (3‐year moving‐average) with linear regression.

### 2.6 Age at maturity

We estimated age at maturity (A_50_, age at 50% probability of the individuals have reached maturation age) for each cohort (year class) with sufficient data in the time series for perch in both Lake Vaggatem (n_cohorts_ = 12) and Lake Skrukkebukta (n_cohorts_ = 4) (Appendix Figure S13) using logistic regression. We related the estimated cohort‐specific A_50_ to the estimated total cohort‐specific length increment (age 1 to 4 years old) using linear regression. We estimated cohort‐specific $A_{50}$ to address how environmental variables (water temperature and relative density) indirectly affected age at maturity mediated through individual juvenile growth. In addition, we estimated maturation age separately for males and females to explore whether it differed between the sexes and whether the proportion of males and females changed over time (Appendix S8, Figures S11 and S12).

Age at maturation is assumed to be plastic and depends on a probabilistic maturation reaction norm (PMRN) describing the length‐ and age‐specific probabilities of maturation (Heino et al., 2002). To illustrate how age at maturity changes with differing individual growth rates and to highlight the population response to altered individual growth rates, we calculated the PMRN from the long‐term data on perch in the Pasvik watercourse (See details on PMRN estimation routine in the Supplementary Information, Appendix S8).

To investigate the causal relationship between environmental variables and age at maturation (A_50_) we used structural equation modeling (SEM) with the “piecewiseSEM” package in R. We constructed the SEM to assess direct and indirect effects of summer water temperature and relative density on age at maturation (A_50_) mediated through mean length increment (mm·year^−1^) from age 1‐ to age 4‐year‐old perch. Summer water temperature and relative density of perch were modeled as exogenous random variables, influencing other variables but not themselves being influenced by other variables. The biotic variable length increment (from age 1 to age 4, mm·year^−1^) was included as an endogenous variable influenced by others and itself also influencing other variables. Finally, age at maturity (A_50_) was set as a response endogenous variable, influenced by all other variables but not influencing other variables. All variables were standardized prior to the analysis. Figures and maps were created by using the ggplot package in R or BioRender.com, and tables were made using the Sjplot package in *R* 4.2.2 with 0.05 significance level.

## 3 RESULTS

### 3.1 The proportion of perch is increasing in high latitude lakes

**FIGURE 2:**
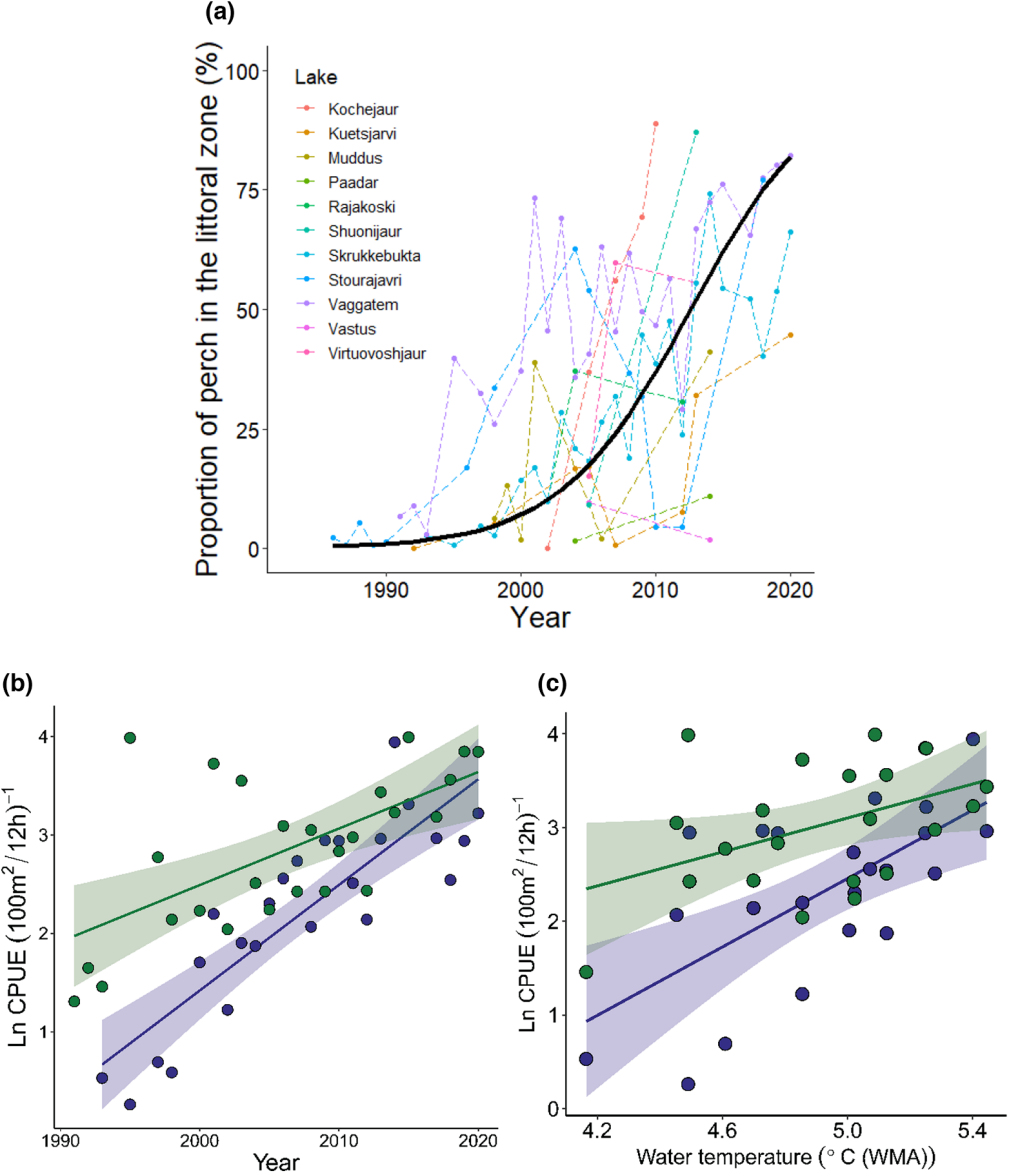
Development of perch populations in the littoral zone of study lakes from year 1990 to year 2020. (a) Proportion of perch in the study lakes, the black line shows the overall trend given by the generalized linear mixed‐effect model (Table S11). (b) Relative density (ln‐CPUE, no. of fish per 100 m^2^ per 12 h) of perch in the littoral zone of Lake Vaggatem (green, *n* = 26) and Lake Skrukkebukta (blue, *n* = 24) from year 1990 to year 2020. (c) Relative density (ln‐CPUE) of perch dependent on annual mean water temperature (weighted‐moving‐average [WMA] over the last 2 years) in Lake Skrukkebukta (blue, *n* = 22) and Lake Vaggatem (green, *n* = 22) in the Pasvik watercourse.

The proportion of perch in the littoral zone of the sampled lakes substantially increased over the study period (Figure 2a). All lakes, with the exception of one which only had two sampling points, showed an overall increase in the proportion of perch with time. The overall mean trend reveals that the proportion of perch increased exponentially over time (*p* < .01, mar‐*R* ^2^ = .459, Table S10), from under 10% in the early 1990s to above 70% in most lakes during the last decade, however, with large variation between lakes (Figure 2a). The relative density (CPUE) data in Lake Skrukkebukta and Lake Vaggatem show a similar trend as the overall proportion data (Figure 2b), with a substantial increase in relative density of perch in the littoral zone of both lakes (Skrukkebukta: *t* = 8.014 on 22 d.f., *p* < .01, adj‐*R* ^2^ = .73. Vaggatem: *t* = 4.042 on 24 d.f., *p* < .01, adj‐*R* ^2^ = .38, Appendix Table S11 and S12). This was related to an increase in water temperature where the relative density of perch increased with annual mean water temperature (weighted‐moving‐average over the last 2 years) in both Lake Skrukkebukta and Lake Vaggatem (Figure 2c). In Lake Skrukkebukta the relative density has increased with 1.83 ln‐CPUE·°C^−1^ of temperature increase (*t* = 3.788 on 20 d.f., *p* < .01, adj‐*R* ^2^ = .389, Appendix Table S13), while in Lake Vaggatem, the increase was weaker but significant with an increase of 0.89 ln‐CPUE·°C^−1^ of temperature increase (*t* = 3.788 on 20 d.f., *p* = .048, adj‐*R* ^2^ = .141, Appendix Table S14). In comparison with the cool‐water adapted perch, the cold‐water adapted species whitefish show a significant decrease in relative density in both lakes throughout the time series (Skrukkebukta: *t* = −2.60 on 22 d.f., *p* = .016, adj‐*R* ^2^ = .20. Vaggatem: *t* = −4.91 on 24 d.f., *p* < .01, adj‐*R* ^2^ = .48, Appendix Figure S14, Tables S27 and S28).

### 3.2 Recruitment of juveniles increases with summer water temperature

**FIGURE 3:**
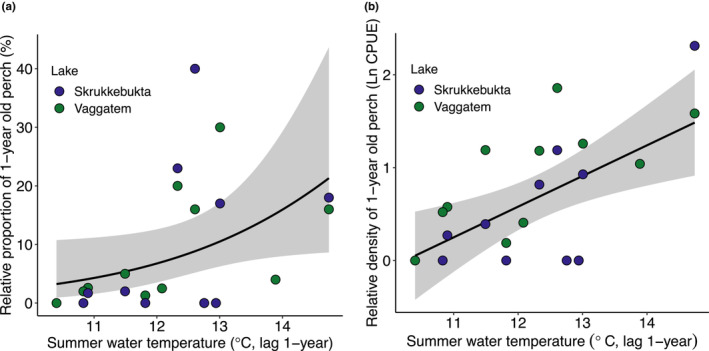
(a) Relative proportion of one‐year‐old individuals in Lake Skrukkebukta (blue) and Lake Vaggatem (green) perch populations (*n* = 23) related to the preceding summer water temperature (°C), the black line shows the overall trend given by the Generalized linear model (Table S15). (b) Relative density of one‐year‐old perch (CPUE, 100 m^2^/12 h)^−1^ in the littoral zone of the two lakes (*n* = 23), the black line shows the overall trend given by the linear model (Table S16).

We used the relative proportion of 1‐year‐olds in the perch population in addition to the relative density of 1‐year‐olds in Lake Vaggatem and Lake Skrukkebukta as a proxy for the number of recruits to the perch population after surviving the 1st critical winter. The relative proportion of 1‐year‐old perch increased with mean summer water temperature (Jun–Aug) the preceding year (Figure 3a) (*p* = .037, McFadden's‐*R* ^2^ = .205, Appendix Table S15). The relative density of 1‐year‐old perch was also significantly related to temperature (Figure 3b) (*p* < .001, adj‐*R* ^2^ = .35), increasing by 3.2% per degree centigrade increment in preceding year summer water temperature (°C) (*t* = 3.550 on 21 d.f., *p* < .001, Appendix Table S16).

### 3.3 Faster juvenile growth with higher summer water temperature

**FIGURE 4:**
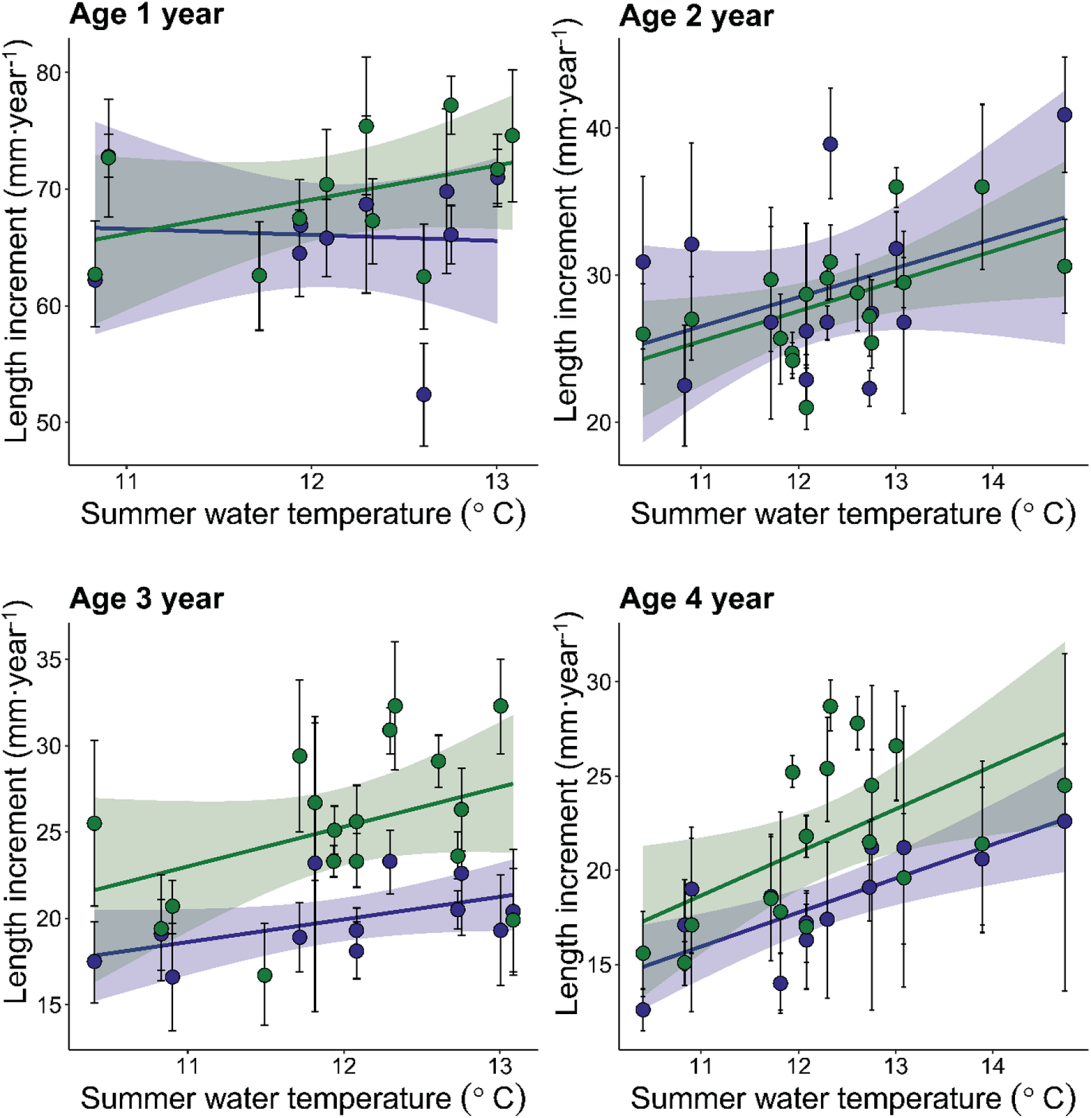
Back‐calculated length increment (mm·year^−1^) for age 1 year (top left, *n* = 699), 2 years (top right, *n* = 1457), 3 years (bottom left, *n* = 1294), and 4 years (bottom right, *n* = 1235) perch from Lake Vaggatem (green) and Lake Skrukkebukta (blue) in the Pasvik watercourse dependent on summer water temperature (°C). Points represent the mean, whiskers represent the bootstrapped 95% confidence interval of the mean, and trend line with shading represents linear regression with standard error.

**FIGURE 5:**
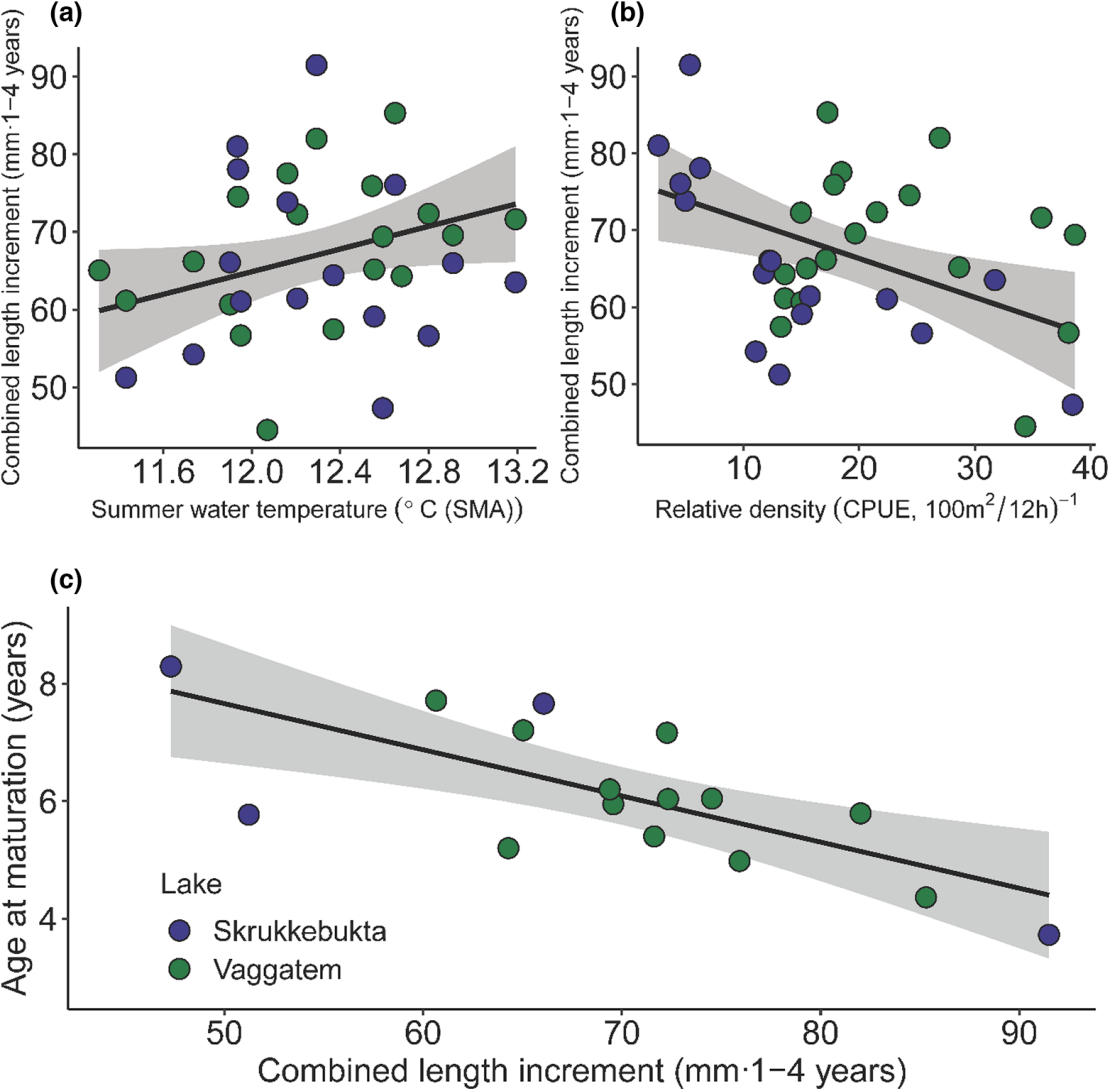
Relationship between the average length increment from age 1 to 4 years (mm·1–4 years^−1^) for the different cohorts of perch (*n* = 35) and (a) summer water temperature (three‐year moving‐average, SMA), (b) relative density (three‐year moving‐average, CPUE) with solid lines representing the multiple linear regression model results (predictors centered and scaled). (c) The relationship between age at maturation (A50, given by logistic regression) and average length increment from age 1 to 4 years for the different perch cohorts from 1998 to 2013 (*n* = 16), with the solid line representing the linear regression model and shaded area depicting the standard error of the model. NB. Lakes were pooled in the linear regressions because no significant difference in the slope between lakes was detected.

The overall trend for the juvenile perch was that annual length increment increased with summer water temperature and decreased with the relative density of perch in both Lake Vaggatem and Lake Skrukkebukta (Figure 4, Appendix Figures S7–S10). The combined length increment (mm·year^−1^) from age 1‐ to age 4‐year‐old perch increased substantially with increasing 3‐year‐mean summer water temperature and decreased similarly with an increase in 3‐year‐mean relative density (Figure 5a,b). The combined length increment (age 1 to 4 year) for Lake Vaggatem and Lake Skrukkebukta perch was significantly related to temperature and density in a linear regression model (*p* = .004, adj‐*R* ^2^ = .28), increasing by 8.5 mm per degree centigrade of temperature increment (*t* = 2.481 on 31 d.f., *p* = .019) and decreased by 6.8 mm per 10 CPUE increment (*t* = −3.806 on 31 d.f., *p* = .001) (Appendix Table S17). In addition, there was a difference in intercept between the lakes, where the length increment was larger in Lake Vaggatem compared with Lake Skrukkebukta (Appendix Table S17). However, for the individual age groups, the effect of water temperature and relative density on length increment varied. For the youngest age group (1 year old) there was no significant change in length increment (mm·year^−1^) with either increasing summer water temperature or relative density of perch (Figure 4, Appendix: Figure S7, Tables S18 and S19). For all the other juvenile age groups (2–4 year old), length increment (mm·year^−1^) increased significantly with increasing summer water temperature in both lakes, whereas only in Lake Vaggatem length increment decreased significantly with the relative density of perch (Figure 4, Appendix: Figures S6–S10, Tables S20–S25) (See Supplementary information for more detailed description of results).

### 3.4 Earlier maturation age with faster growth

**FIGURE 6:**
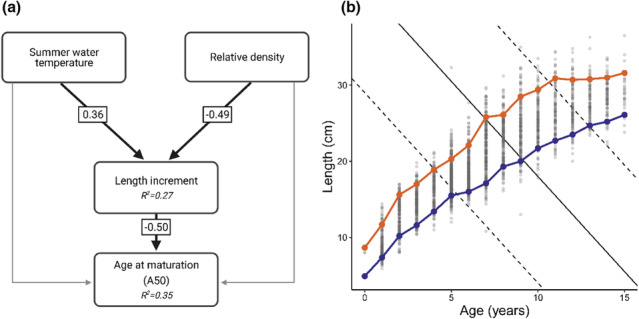
(a) Structural equation model showing the relationship between predictor variables affecting length increment (combined from age 1‐year to age 4‐year‐olds) and the effect of increasing length increment on age at maturation in the perch populations in Lake Vaggatem and Lake Skrukkebukta. Arrows represent causal pathways, highlighted black lines represent significant relationships and gray lines represent nonsignificant relationship within the model. Numbers in boxes denote the effect size of standardized coefficients and *R* ^2^ is shown for each endogenous variable. (b) Relationship between somatic growth and maturation in perch in the Pasvik watercourse illustrated by the maturation reaction norm, length at age for perch in the Pasvik watercourse (gray dots) with blue dotted line representing a slow growth rate, that is, “cold and high‐density situation” (10th percentile) and orange dotted line representing a fast growth rate, that is, “warm and low‐density situation” (90th percentile). The population estimated probabilistic maturation reaction norm (PMRN) midpoint (solid line), the 25th and 75th percentile (dashed lines).

Age at maturation (A_50_) differed between males and females in the perch populations in Lake Vaggatem and Lake Skrukkebukta; males matured on average at an age of 4.1 years whereas females matured on average at an age of 7.5 years (Appendix Figure S11). The difference in age at maturity between the sexes did not change over time (*t* = −.767 on 12 d.f., *p* = .45) (Appendix Figure S12). The observed increase in combined length increment (mm·1‐4 years^−1^) substantially affected the cohort‐specific age at maturation (A_50_) negatively (Figure 5c), with −0.8 years reduction per cm increase in length increment (*t* = −3.783 on 14 d.f., *p* = .002, adj‐*R* ^2^ = .47) (Appendix Table S25 and Figure S13). The effect of summer water temperature and relative density on age at maturity was mediated through growth (length increment) for perch in Lake Vaggatem and Lake Skrukkebukta, as illustrated by the structural equation model (SEM) results (Figure 6a). We found a positive effect of cohort‐specific (age 1 to age 4 year old) mean summer water temperature and a negative effect of relative density of perch in the same time period on length increment of perch from age 1 to age 4 years, which further affected age at maturity (A_50_) negatively (Figure 6a). Figure 6b illustrates these relationships theoretically, where individuals with higher growth rates, due to temperature increase or reduced density, will reach maturation age earlier than individuals experiencing lower growth rates according to the estimated PMRN from the perch populations in Lake Vaggatem and Lake Skrukkebukta.

## 4 DISCUSSION

We find that the numerical importance of perch in fish communities at the northern edge of its distribution increased substantially during the last three decades of rapid warming. The positive trend was registered in ten out of the eleven lakes investigated, whereas the outlier lake had a relatively low contribution of perch in both sampling periods. For our main two study lakes, Lake Vaggatem and Lake Skrukkebukta, the trend was driven by a significant increase in perch density associated with the increase in water temperature. These two perch populations showed higher recruitment with warming, with the relative proportion of 1‐year‐old fish in catches increasing significantly with an increment in mean summer water temperature. The demographic responses to warming were concomitant with individual‐level effects on somatic growth, which increased with temperature across most young age classes, resulting in earlier maturation. The latter is an indirect effect of warming mediated by the increased temperature‐dependent growth rate of juveniles, an ecophysiological response, and phenotypic plasticity in maturation schedules, a life‐history adaptation. The resulting earlier maturation and larger size at the age of juveniles help explain the increased recruitment rates promoting perch population density at higher temperatures. Somatic growth displayed negative density‐dependence, which may mask individual and demographic responses to warming in field studies.

During the recent period of warming, the increased numerical importance of perch was accompanied by positive temperature effects on juvenile growth in our two reference lakes. The yearly mean growth rate significantly increased with temperature across all investigated juvenile age classes, with the exception of the 1‐year‐old age group. The cohort mean cumulative length increment from age 1 to 4 years increased by 8.5 mm (~12%) per degree centigrade increment in summer water temperature. Individual perch growth displayed substantial negative density‐dependence, decreasing by 6.8 mm per 10 CPUE units increase in relative density, in line with earlier findings for this and other species (Byström & García‐Berthou, 1999; Olin et al., 2017). The observed positive effect of temperature on perch somatic growth was expected considering that in our lakes mean summer water temperature varied between 10 and 14°C, which, although within the species temperature tolerance range (Karås & Thoresson, 1992), is well below the optimum temperature for perch growth, estimated to be within 16–27°C (Hokanson, 1977). Positive effects of higher summer water temperatures on perch growth rates have been described in regions where temperature variability is within the thermal tolerance range of the species (Huss et al., 2019; Jeppesen et al., 2012), where the increased size at age was maintained also in adult age classes owing to faster growth in young stages (Gårdmark & Huss, 2020; Huss et al., 2019). The projected future increase in ambient temperatures will increase metabolic demands (Huey & Kingsolver, 2019), but at high latitudes, lake productivity mediated by catchment greening is expected to increase with warming (O'Beirne et al., 2017) and should ensure sufficient food availability to support growth (Kao et al., 2015). The documented and projected positive effects of warming on the growth of perch living at its northern range edge affect its life‐history, demography, and ecological interactions. The faster growth induced by warming resulted in earlier maturation of perch. The indirect effect of warming, estimated and summarized via a structural equation model, is substantial, with maturation age (A_50_) decreasing by 0.8 years per cm increase in juvenile length increment (from age 1 to 4 years). The adaptive plastic response in maturation age is dependent on the ecophysiological process of somatic growth, which in turn is affected by the ambient temperature and food availability (Ward et al., 2017). A reduction in maturation age as a consequence of increased growth has been documented repeatedly in fish (Haugen, 2000; Reznick, 1993).

We observed an increase in both density of recruits (1‐year‐olds) and proportion of 1‐year‐olds with summer water temperature in our two main study lakes. Increasing summer water temperatures has been shown to increase recruitment in cool‐water fish (Kokkonen et al., 2019; Svärdson & Molin, 1968). An increase in recruitment could reflect either an increase in reproductive output at the population level, faster population turnover rate, that is, higher relative mortality of adults and/or a decrease in mortality of eggs, hatchlings, and juveniles. Furthermore, an increase in juvenile growth rate has been associated with a subsequent increase in reproductive output in fish (Ward et al., 2017), and an increase in reproductive output is seen as a direct effect of an increase in water temperature also for perch (Heibo et al., 2005). The climate‐driven increase in temperature‐dependent growth thus results in larger size at age and earlier maturation, increasing the perch population reproductive output and recruitment, thus promoting population growth, as seen in other stocks (Ward et al., 2017). Survival of individuals during different life stages is a process, which could be directly affected by ambient temperature either through temperature‐specific developmental rates, temperature‐dependent mortality rates, or time spent in different life stages mediated by individual somatic growth (Mirth et al., 2021; Sponaugle et al., 2006). The effect of increased summer temperature is usually related to larger body size and condition in autumn that subsequently lower the winter mortality (Estlander et al., 2017; Hurst, 2007), but a boosted growth was not evident in our dataset for the 0+ age class. However, mortality and the duration of the perch egg stage are decreasing with temperature, with normal development of eggs occurring in the temperature range of 7–18°C (Küttel et al., 2002). Therefore, an increase in summer water temperature might increase the number of surviving hatchlings as more eggs might develop normally and the shorter duration of the egg stage might decrease the predation risk at this life stage. Embryos and hatchlings are defined as critical life stages with a narrow thermal range (Dahlke et al., 2020) and at the northern edge of perch distribution an increase in spring and summer water temperature might have been pivotal for an increase in recruitment and subsequent increase in density and relative importance of the perch populations. Changed mortality schedules, larger size at age, and younger age at maturity with increased water temperature might all be important contributors to the observed increase in recruitment of 1‐year‐olds in the perch populations.

Considering the rapid warming experienced in the study area during the last three decades, an increased numerical importance of a cool‐water species could be expected (Ficke et al., 2007; Rolls et al., 2017), but a total shift in fish community dominance was surprising. The population process outlined above helps explain the sudden response to climate warming and suggests that similar responses should be expected in other populations of cool‐water species at their northern range edge unless kept in check by negative ecological interactions. However, many of the lakes near the northern range edge of perch are salmonid‐dominated systems, with little resistance capacity against percid fish at higher temperatures (Hayden et al., 2014). Here, we demonstrated that the increase in the relative density of the cool‐water perch was accompanied by a decrease in the relative abundance of the cold‐water adapted whitefish in the littoral zone. Shifts in dominance from cold‐water fish to more cool‐ or warm‐water fish have been documented in other freshwater systems as well (Hansen et al., 2017; Jeppesen et al., 2012). Climate changes impact on high latitude freshwater ecosystems is predicted to further increase as warming favors resident and invasive cool‐water species, potentially displacing native cold‐water salmonids from these ecosystems (Campana et al., 2020; Hansen et al., 2017; Hayden et al., 2017). Perch is a generalist fish that has high capacity as a resource competitor in littoral habitats, and subsequently also as a predator, for native cold‐water species such as whitefish (Hayden et al., 2014). Such ecological interactions with resident cold‐water species, mediating higher‐order effects of climate change, may change in character and outcome under warming (Urban et al., 2011). Climate‐induced changes in size‐structured interactions may have cascading effects within the food web, and the outcome is dependent on thermal niche, population size structure, and the existing ecological interactions within the ecosystem (Gårdmark & Huss, 2020). Cold‐water sympatric species will be more vulnerable as cool‐water fish increase in competitive and predatory capacity with warming, possibly causing major alterations within fish communities in high latitude lakes. In a wider perspective, cool‐water perch dominance may eventually shift towards warm‐water cyprinid fish (roach, *Rutilus rutilus*, and bleak, *Alburnus alburnus*) dominance along with increasing temperature and productivity in lakes where cyprinids are present or able to immigrate (Hayden et al., 2017).

## 5 CONCLUSION

Our study documents that warming triggers a rapid numerical increase of cool‐water fish at their northern range edge. The causal links between individual and population effects of warming considered in this study help to account for the speed and magnitude of the population responses. The magnitude of these responses is such that dominance is shifting from salmonids to percids, constituting a significant warning of an ongoing reorganization of high latitude fish communities. Evidently, water‐temperature increase from climate change has already favored cool‐water fish at high latitudes, and future projected climate warming will accentuate this development, potentially at the further expense of cold‐water salmonids. Climate adaptation strategies must therefore focus on limiting the ecological impact of warmer water fish in high latitude ecosystems. Consequently, management should focus on climate adaptation limiting future introductions and invasions of cool‐water fish and mitigating harvesting pressure on cold‐water fish.

## AUTHOR CONTRIBUTIONS

**Aslak Smalås:** Conceptualization (lead); data curation (supporting); formal analysis (lead); methodology (lead); project administration (supporting); visualization (lead); writing – original draft (lead). **Raul Primicerio:** Conceptualization (lead); data curation (supporting); formal analysis (supporting); methodology (supporting); project administration (lead); visualization (supporting); writing – original draft (supporting). **Kimmo K Kahilainen:** Conceptualization (supporting); data curation (lead); formal analysis (supporting); methodology (supporting); project administration (supporting); visualization (supporting); writing – original draft (supporting). **Petr Terentyev:** Conceptualization (supporting); data curation (lead); formal analysis (supporting); methodology (supporting); project administration (supporting); visualization (supporting); writing – original draft (supporting). **Nikolay Kashulin:** Conceptualization (supporting); data curation (lead); formal analysis (supporting); methodology (supporting); project administration (supporting); visualization (supporting); writing – original draft (supporting). **Elena Zubova:** Conceptualization (supporting); data curation (supporting); formal analysis (supporting); methodology (supporting); project administration (supporting); visualization (supporting); writing – original draft (supporting). **Per‐Arne Amundsen:** Conceptualization (lead); data curation (lead); formal analysis (supporting); methodology (supporting); project administration (lead); visualization (supporting); writing – original draft (supporting).

## FUNDING INFORMATION

This research received funding from multiple sources, including the Academy of Finland (Grant No. 1268566), the Finnish Ministry of Agriculture and Forestry (Grant No. 140903), the H2020 Food Program (Grant No. 677039), the Research Council of Norway (Norges Forskningsråd) (Grant No. 183984), the RSF (Russian Science Foundation) (Grant No. 19‐77‐10007), and the Russian Foundation for Basic Research (Grant No. 18‐05‐60125). We would like to express our sincere gratitude to these funding agencies for their financial support, which was instrumental in conducting this research.

## Supporting information


Appendix S1:

## Data Availability

The data that support the findings of this paper are available at a Dryad‐repository (https://doi.org/10.5061/dryad.tqjq2bw4f).
