# Supplementary material for: Increased importance of cool‐water fish at high latitudes emerges from individual‐level responses to warming
Source: Ecol Evol. 2023 Jun 6;13(6):e10185. doi: 10.1002/ece3.10185 (PMC10244614; doi:10.1002/ece3.10185)
Supplement: Supplementary file 1 — Appendix S1: [file ECE3-13-e10185-s001.docx]

**Supplementary information**

**Appendix 1: Description of the study species (Eurasian perch), conceptual model of mechanisms, lake characteristics and summary data of the sampled material**

The Eurasian perch, *Perca fluviatilis*, is distributed throughout northern Eurasia, ranging from 40°N to 70°N. The species is common and widespread in lakes, ponds and slow-running rivers. It is a generalist that has a size-dependent ontogenetic dietary niche-shift, where larvae and small juveniles are pelagic zooplankton feeders, before they shift to feeding on benthic macroinvertebrates at intermediate sizes, and larger individuals feed on fish (Persson 1988, Hjelm et al. 2001, Amundsen et al. 2003). It is common in both recreational and subsistence fisheries, with a recorded global catch of around 30 000 tons (FAO 2020). Perch is a cool-water fish (e.g. Shuter et al. 2012, Hayden et al. 2014), with temperature requirements for development and growth that are size and stage dependent (Hokanson 1977, Dahlke et al. 2020). The temperature range for normal egg development is between 7-18 ˚C, and the optimum is estimated to be between 13-14˚C (Hokanson & Kleiner 1974, Hoestlandt & Devienne 1980, Saat et al. 1996). The reported minimum temperature for growth is between 5-10 ˚C (Karås 1987, Karås 1990, Hokanson & Kleiner 1974) and maximum temperature for growth between 31.4- 33.5 ˚C (Alabaster & Downing 1966, Willemsen 1977). Optimal temperature ranges for growth vary between the different life stages. At the larval stage there is an optimum range between 12-25 ˚C (EIFAC 1969 in Küttel et al. 2002, Karås 1996), whereas for juveniles the reported optimum temperature is 25 ˚C (Hokanson & Kleiner 1974). For adult perch, the reported optimum range is between 16-27 ˚C (Horoszewicz 1973 in Hokanson 1977, Craig 1978), and the range of spawning temperatures falls between 5-19 ˚C (Hokanson 1977) (see Appendix Fig. S1 for relationship between water temperature and somatic growth in perch).


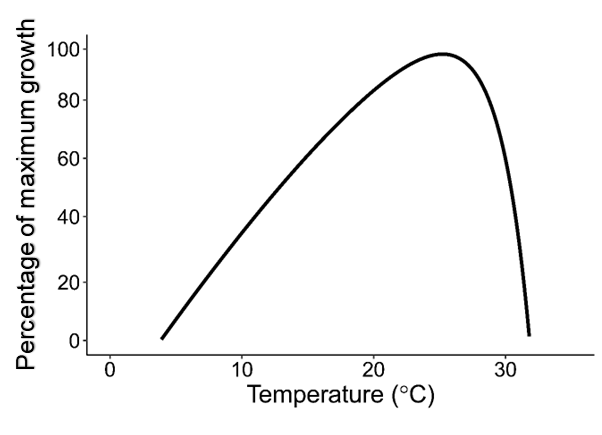


Figure S1. The relationship between growth and water temperature for perch fed on maximum rations from existing literature. Where minimum temperature for growth is 5 °C, optimum temperature for growth is 25 °C and maximum temperature for growth is 32 °C.


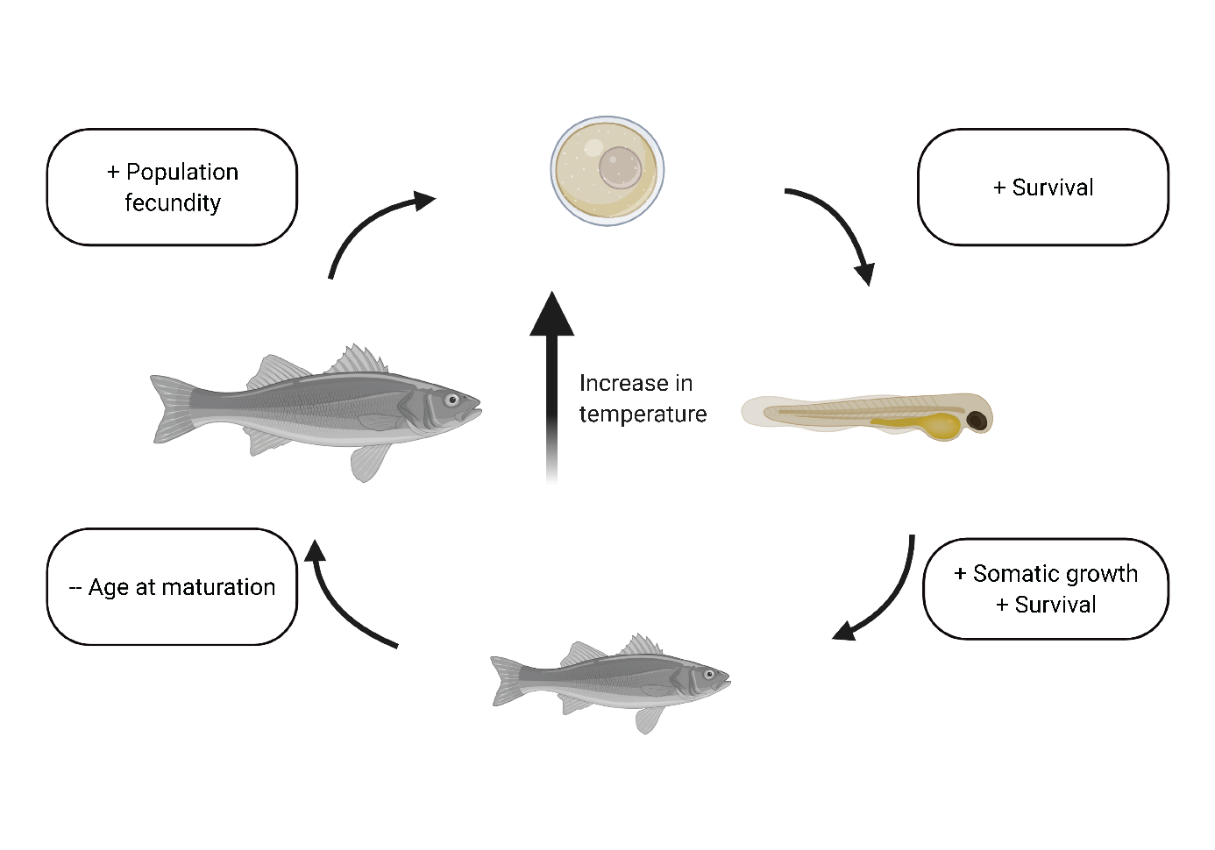


Figure S2. Schematic representation of how the life cycle of the Eurasian perch might be affected by increased water temperatures in high latitude populations.


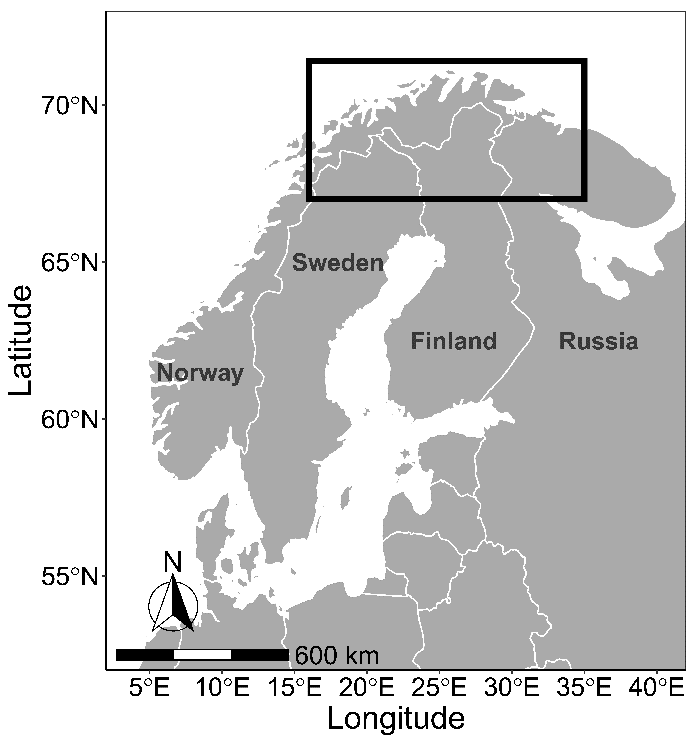

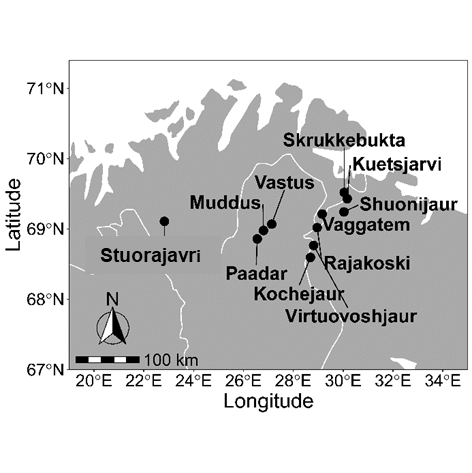


Figure S3**.** Map of northern Europe with the box depicting the study area (left panel), and the location of the sampled lakes within the study area (right panel).


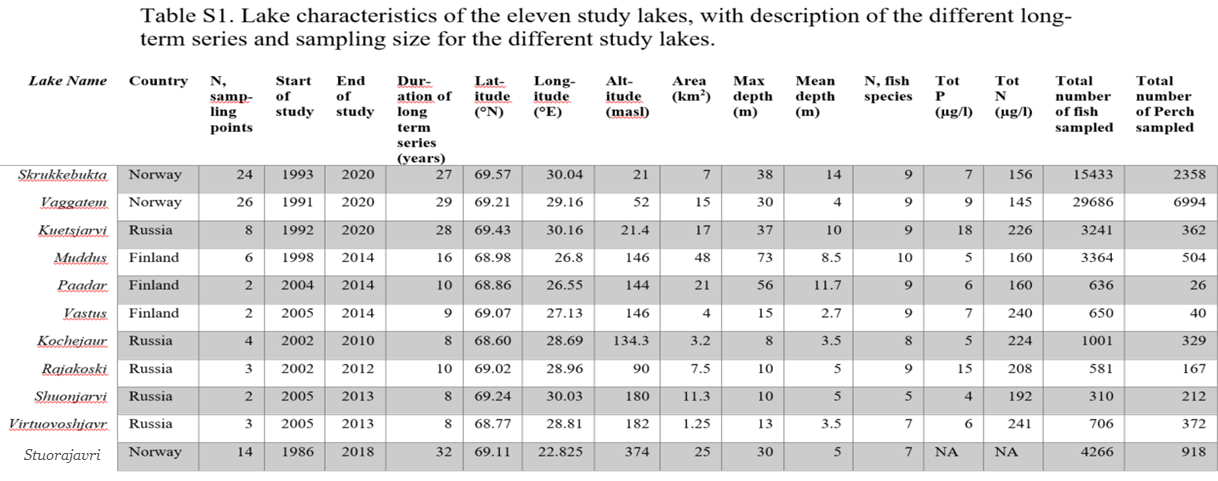


The sampled lakes varied in fish community composition. In addition to perch, European whitefish (*Coregonus lavaretus*) was present in all lakes except for Lake Shuonijaur and was either the most abundant or the second most abundant fish species, next to perch, in the littoral zone of the different lakes. Besides perch and whitefish, pike (*Esox lucius*), burbot (*Lota lota*), brown trout (*Salmo trutta*), grayling (*Thymallus thymallus*), nine-spined stickleback (*Pungitius pungitius*) and common minnow (*Phoxinus phoxinus*) were present in lower abundances in most lakes. Arctic charr (*Salvelinus alpinus*) was only present in Lake Shuonijaur and Lake Stuorajavri, while vendace (*Coregonus albula*) was present in a few lakes.

Table S2. Summary data per sampling year for perch in Lake Vaggatem including total number of perch (n), mean body length (cm), mean body weight (g), proportion of female perch in the dataset, mean condition factor and the von Bertalanffy`s growth variables $L_{\infty}$ and initial growth, $g$. NA describes missing data.

|  | Number of fish | Mean body length (cm) | Mean body weight (g) | Proportion female perch | Proportion of one-year olds | Mean Condition factor | Asymptotic length, $\boldsymbol{L}_{\boldsymbol{\infty}}$ | Initial growth, $\boldsymbol{g}$ (cm·year^-1^) |
| --- | --- | --- | --- | --- | --- | --- | --- | --- |
| 2003 | 608 | 19.7 | 139.8 | 0.56 | 0.16 | 1.32 | 31.47 | 6.0255 |
| 2004 | 55 | 18.1 | 98.8 | 0.6 | 0.20 | 1.26 | 26.47 | 8.059 |
| 2005 | 132 | 21.1 | 165.1 | 0.67 | 0.30 | 1.33 | 26.89 | 8.67 |
| 2006 | NA | NA | NA | NA | NA | NA | NA | NA |
| 2007 | NA | NA | NA | NA | NA | NA | NA | NA |
| 2008 | 110 | 19.5 | 110.8 | 0.58 | 0.025 | 1.25 | 25.62 | 6.31 |
| 2009 | 109 | 23.4 | 202.7 | 0.63 | 0 | 1.39 | 36.22 | 5.13 |
| 2010 | 86 | 21.1 | 134.6 | 0.65 | 0.013 | 1.31 | 29.03 | 5.257 |
| 2011 | NA | NA | NA | NA | NA | NA | NA | NA |
| 2012 | NA | NA | NA | NA | NA | NA | NA | NA |
| 2013 | 38 | 22.5 | 192.9 | 0.68 | 0.026 | 1.37 | 30.35 | 6.02 |
| 2014 | 165 | 19.7 | 142.7 | 0.64 | 0.16 | 1.29 | 27.86 | 5.99 |
| 2015 | 121 | 20.6 | 153.4 | 0.69 | 0 | 1.32 | 30.77 | 5.64 |
| 2016 | NA | NA | NA | NA | NA | NA | NA | NA |
| 2017 | 181 | 20.6 | 132.1 | 0.65 | 0 | 1.26 | 27.44 | 6.83 |
| 2018 | 100 | 20.5 | 139.0 | 0.65 | 0.02 | 1.31 | 29.9 | 4.92 |
| 2019 | 158 | 19.5 | 118.2 | 0.54 | 0.04 | 1.29 | 30.57 | 4.58 |
| 2020 | 172 | 18.6 | 103.4 | 0.54 | 0.05 | 1.28 | 26.95 | 5.32 |

Table S3. Summary data per sampling year for perch in Lake Skrukkebukta including total number of perch (n), mean body length (cm), mean body weight (g), proportion of female perch in the dataset, mean condition factor and the von Bertalanffy`s growth variables $L_{\infty}$ and initial growth, $g$. NA describes missing data. NA* describes data which were not good enough to estimate the different variables.

|  | Number of fish | Mean body length (cm) | Mean body weight (g) | Proportion female perch | Proportion of one-year olds | Mean Condition factor (K) | Asymptotic length, $\boldsymbol{L}_{\boldsymbol{\infty}}$ | Initial growth, $\boldsymbol{g}$ (cm·year^-1^) |
| --- | --- | --- | --- | --- | --- | --- | --- | --- |
| 2003 | 30 | 16.0 | 63.6 | 0.53 | 0.40 | 1.20 | NA* | NA* |
| 2004 | 50 | 15.6 | 57.8 | 0.62 | 0.23 | 1.20 | NA* | NA* |
| 2005 | 73 | 15.0 | 50.1 | 0.53 | 0.17 | 1.17 | NA* | NA* |
| 2006 | NA | NA | NA | NA | NA | NA | NA | NA |
| 2007 | NA | NA | NA | NA | NA | NA | NA | NA |
| 2008 | NA | NA | NA | NA | NA | NA | NA | NA |
| 2009 | NA | NA | NA | NA | NA | NA | NA | NA |
| 2010 | 67 | 17.1 | 63.4 | 0.70 | 0 | 1.18 | 22.59649 | 5.55072 |
| 2011 | NA | NA | NA | NA | NA | NA | NA | NA |
| 2012 | NA | NA | NA | NA | NA | NA | NA | NA |
| 2013 | 59 | 23.2 | 197.4 | 0.66 | 0.02 | 1.33 | 37.84762 | 4.326264 |
| 2014 | 130 | 19.2 | 121.1 | 0.62 | 0.18 | 1.20 | 24.26648 | 9.368055 |
| 2015 | 47 | 19.2 | 136.2 | 0.57 | 0 | 1.15 | 36.95272 | 4.695531 |
| 2016 | NA | NA | NA | NA | NA | NA | NA | NA |
| 2017 | 78 | 20.4 | 148.3 | 0.54 | 0 | 1.20 | 29.62441 | 6.796229 |
| 2018 | 81 | 21.2 | 164.7 | 0.64 | 0 | 1.24 | 33.10267 | 4.713568 |
| 2019 | 111 | 20.4 | 133.8 | 0.52 | 0 | 1.17 | 37.64752 | 4.2 |
| 2020 | 100 | 19.8 | 119.3 | 0.59 | 0.02 | 1.22 | 31.75967 | 4.725684 |

**Appendix 2: Water temperature data**

Table S4. Summary results for the linear model of the development of mean annual water temperature over the sampling period (year 1990 to year 2020).

|  | **Mean Annual Water temperature** | | |
| --- | --- | --- | --- |
| *Predictors* | *Estimates* | *CI* | *p* |
| (Intercept) | 4.42 | 4.07 – 4.78 | **<0.001** |
| year | 0.03 | 0.01 – 0.05 | **0.005** |
| Observations | 27 | | |
| R^2^ / R^2^ adjusted | 0.272 / 0.243 | | |

Table S5. Summary results for the linear model of the development of mean summer water temperature over the sampling period (year 1990 to year 2020).

|  | **Mean Summer Water temperature** | | |
| --- | --- | --- | --- |
| *Predictors* | *Estimates* | *CI* | *p* |
| (Intercept) | 11.27 | 10.42 – 12.12 | **<0.001** |
| year | 0.05 | 0.00 – 0.09 | **0.037** |
| Observations | 27 | | |
| R^2^ / R^2^adjusted | 0.163 / 0.130 | | |

Table S6. Summary results for the linear model of the development of mean autumn water temperature over the sampling period (year 1990 to year 2020).

|  | **Mean Autumn Water temperature** | | |
| --- | --- | --- | --- |
| *Predictors* | *Estimates* | *CI* | *p* |
| (Intercept) | 5.41 | 4.85 – 5.97 | **<0.001** |
| year | 0.05 | 0.02 – 0.08 | **0.002** |
| Observations | 28 | | |
| R^2^ / R^2^ adjusted | 0.302 / 0.275 | | |

**Appendix 3: Population level estimates of somatic growth**

To compare somatic growth in the perch population over time and between the different populations we used, in addition to a back-calculation routine, a modified version of the von Bertalanffy growth model for every sampling year in Lake Skrukkebukta and Lake Vaggatem:

$L\left( t \right)=L_{\infty}-\left( L_{\infty}-L_{0} \right) e^{[-\left( g{L_{\infty}}^{-1} \right)t]}$

where $L\left( t \right)$ is the mean length at age $\left( t \right)$, $L_{\infty}$ is the asymptotic length as age approaches infinity, $L_{0}$ is the length at hatching, $g$ is the absolute initial growth rate (length·year^-1^) which represent the maximum growth rate, occurring early in life according to the von Bertalanffy growth model (Sandlund et al. 2013). The advantage of using the modified version of the von Bertalanffy growth model is that we are not dependent on the unit-less coefficient $k$, which is hard to interpret and is not independent of $L_{\infty}$. In addition, $g$ has the unit length·year^-1^ which is interpretable biologically as opposed to $k$ (Mooij et al. 1999, Sandlund et al. 2013). $L_{\infty}$ and $g$ were estimated using non-linear least-square regression based on length at age data for the different years and populations. Perch hatching size is around 5 mm (Olin et al. 2012) $L_{0}$ were therefore set to 5 mm. To investigate the development in initial growth ($g$, length·year^-1^), we used the summer water temperature (°C) the preceding year and relative density of perch (CPUE) the preceding year (because of the lack of 0+ perch in the data set, we had to relate initial growth to 1 year old fish and used the temperature when these fish were 0 year old) in a multiple linear model with an interaction term between the predictors.

The absolute initial growth ($g$) derived from population estimates of the von Bertalanffy growth model from Lake Skrukkebukta and Lake Vaggatem varied between 4.2 and 9.4 (cm·year^-1^) over the study period (Fig. S4). In a simple linear regression combined for both lakes the initial growth is increasing with 0.6 cm·year^-1^ per degree of increasing summer water temperature the preceding year (t=2.653 on 18 d.f., adj-R^2^=0.24, p=0.016, Appendix Table S7) (Fig. S4). In a multiple regression model with the preceding summer water temperature (scaled and standardized) and relative density (CPUE) of perch (scaled and standardized) there was a large effect of temperature on initial growth rate ($g$) (1.07 cm·year^-1^ per standard deviation of temperature increase) (t=4.397 on 14 d.f., p<0.001, adj-R^2^=0.51), no significant effect of relative density (t=-1.024 on 14 d.f., p=0.32), but a substantial negative effect of the interaction between the two predictors (-1.02 decrease per increase in standard deviation of relative density) (t=-2.940 on 14 d.f., p=0.011 Appendix Table S8).


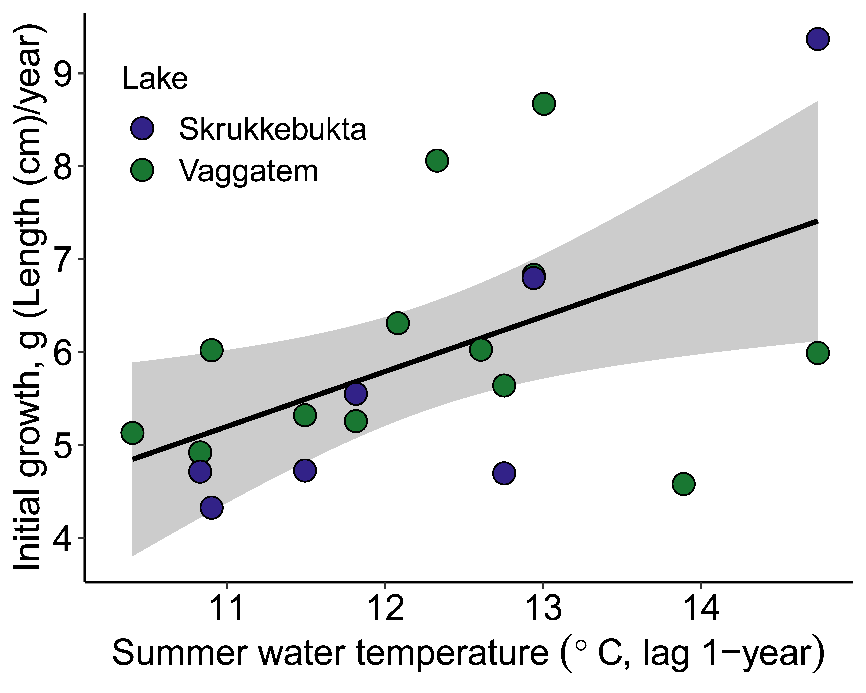


Figure S4. Initial growth (g) given by the modified von Bertalanffy growth model for perch in Lake Skrukkebukta (blue) and Lake Vaggatem (green) dependent on summer water temperature (°C) from the preceding year with a linear regression (solid line) and standard error of the linear regression (shaded area) (n=20).

Table S7. Initial growth (g) (length, cm/year) given by the modified von Bertalanffy growth model in Lake Skrukkebukta and Lake Vaggatem perch populations dependent on mean summer-water-temperature (°C, Jun-Aug) the preceding year in linear regression model.

|  | **Initial growth (g)** | | |
| --- | --- | --- | --- |
| *Predictors* | *Estimates* | *CI* | *p* |
| (Intercept) | -1.30 | -7.07 – 4.47 | 0.809 |
| Preceding year, mean summer-water-temperature (°C, Jun-Aug) (lag-SWT) | 0.59 | 0.12 – 1.06 | **0.016** |
| Observations | 20 | | |
| Degrees of freedom | 18 | | |
| R^2^ / R^2^ adjusted | 0.281 / 0.241 | | |

Table S8. Initial growth (g) (length, cm/year) given by the modified von Bertalanffy growth model in Lake Skrukkebukta and Lake Vaggatem perch populations dependent on mean summer-water-temperature (°C, Jun-Aug) and relative density (CPUE) the preceding year in a linear model with an interaction term of the predictors (standardized and scaled).

|  | **Initial growth (g)** | | |
| --- | --- | --- | --- |
| *Predictors* | *Estimates* | *CI* | *P* |
| (Intercept) | 6.12 | 5.55 – 6.69 | **<0.001** |
| Preceding year, mean summer-water-temperature (°C, Jun-Aug) (lag-SWT) | 1.07 | 0.55 – 1.60 | **0.001** |
| Lag-CPUE_perch_ (lag=1, (100m^2^/12h)^-1^) | -0.30 | -0.93 – 0.33 | 0.323 |
| Lag-SWT * Lag-CPUE_perch_ | -1.02 | -1.77 – -0.28 | **0.011** |
| Observations | 18 | | |
| Degrees of freedom | 14 | | |
| R^2^ / R^2^ adjusted | 0.595 / 0.508 | | |

**
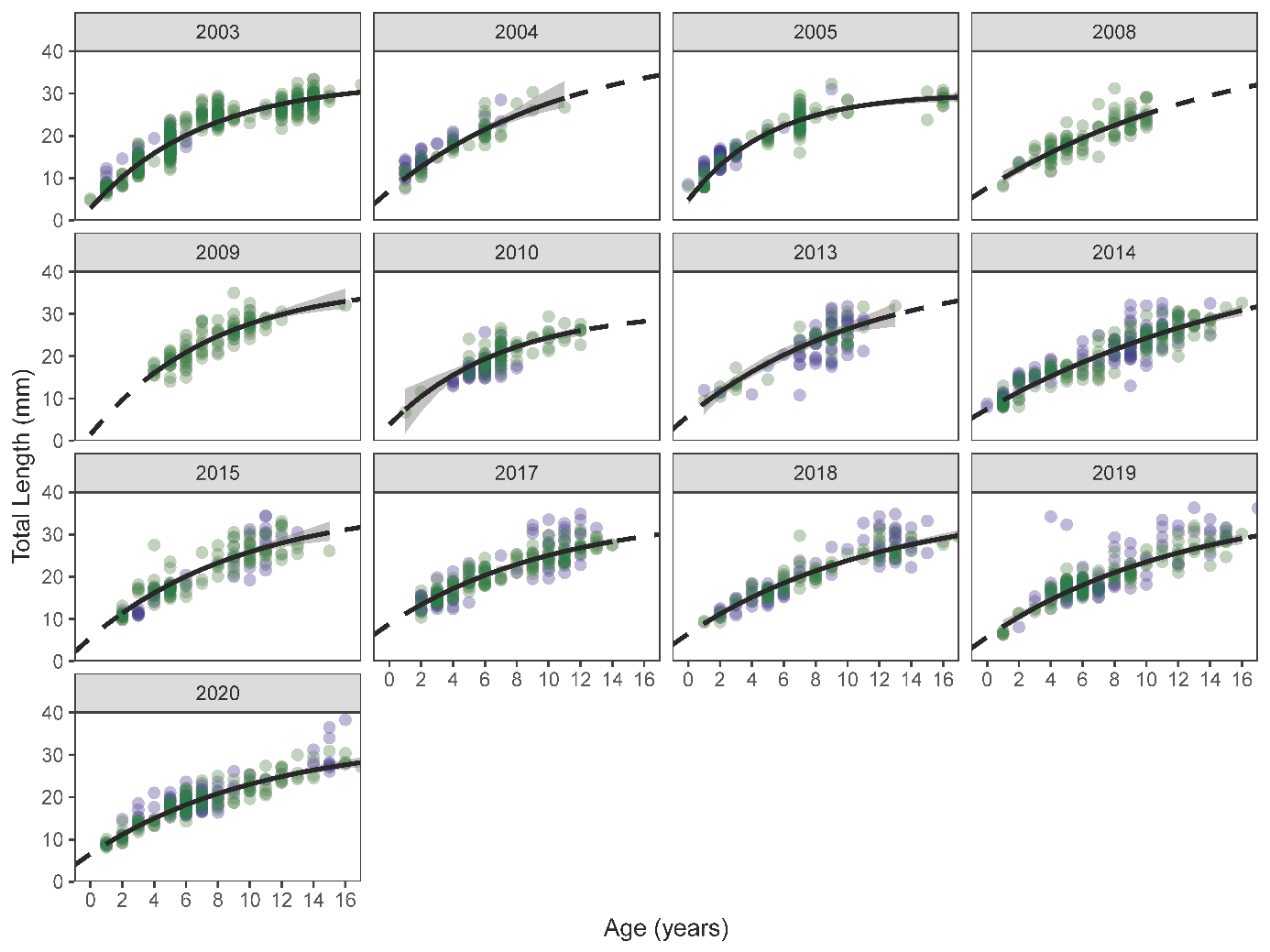
**

Figure S5. Length at age with von Bertalanffy growth model (stippled and solid line) from year 2003 to year 2020 in Lake Skrukkebukta (blue dots) and Lake Vaggatem (green dots). Shaded area represents the bootstrapped 95 % confidence interval of the line.

**Appendix 4: Correlation between back-calculated length and observed length**

Table S9. Linear model representing the correlation between back-calculated length (mm) the final winter before capture and observed length (mm) at capture for perch in both Lake Skrukkebukta and Lake Vaggatem.

|  | **Observed length (mm)** | | |
| --- | --- | --- | --- |
| *Predictors* | *Estimates* | *CI* | *p* |
| (Intercept) | -20.60 | -23.20 – -18.01 | **<0.001** |
| Back-calculated length (mm) | 1.04 | 1.03 – 1.05 | **<0.001** |
| Observations | 1646 | | |
| R^2^ / R^2^ adjusted | 0.938 / 0.938 | | |


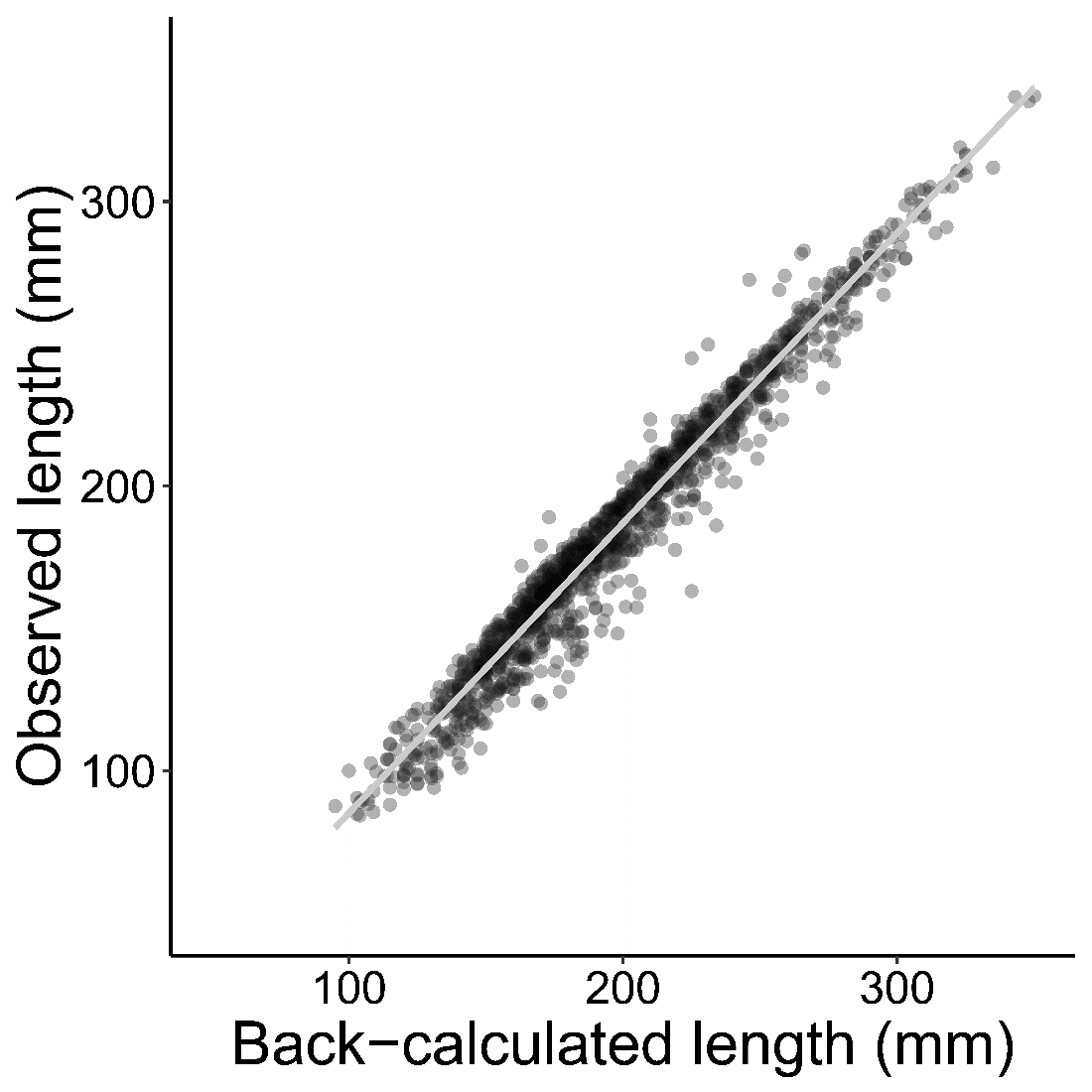


Figure S6. The correlation between back-calculated length (mm) the final winter before capture and observed length (mm) at capture with the solid line (grey) representing the linear regression model (n=1646).

**Appendix 5: Proportion and density of perch in the different study lakes**

Table S10. Summary results for the Generalized Linear Mixed-Effect model (GLMM, with family Binomial) of the development of proportion of Eurasian perch in the littoral zone of 11 high latitude lakes over time.

|  | **Proportion perch** | | |
| --- | --- | --- | --- |
| *Predictors* | *Estimates* | *Std. Error* | *p* |
| (Intercept) | -5.39 | 1.25 | **<0.001** |
| Sampling year | 0.23 | 0.05 | **<0.001** |
| **Random Effects** | | | |
| σ^2^ | 3.29 | | |
| τ_00_ _Lakes_ | 0.49 | | |
| N _Lakes_ | 11 | | |
| Observations | 95 | | |
| Marginal R^2^ / Conditional R^2^ | 0.459 / 0.53 | | |
|  |  | | |

Table S11. Summary results for the linear model of the development in relative density (CPUE) of Eurasian perch in the littoral zone of Lake Skrukkebukta over the 30-year sampling period. The response is on a natural logarithmic scale.

|  | **Ln CPUE Perch (100m^2^/12h)^-1^** | | | |
| --- | --- | --- | --- | --- |
| *Predictors* | *Estimates* | *CI* | *t-value* | *p* |
| (Intercept) | 0.35 | -0.18 – 0.87 | 1.361 | 0.187 |
| Sampling year | 0.11 | 0.08 – 0.14 | 8.014 | **<0.001** |
| Observations | 24 | | | |
| Degrees of freedom | 22 | | | |
| R^2^ / R^2^ adjusted | 0.745 / 0.733 | | | |

Table S12. Summary results for the linear model of the development relative density (CPUE) of Eurasian perch in the littoral zone of Lake Vaggatem over the 30-year sampling period. The response is on a natural logarithmic scale.

|  | **Ln CPUE Perch (100m^2^/12h)^-1^** | | | |
| --- | --- | --- | --- | --- |
| *Predictors* | *Estimates* | *CI* | *t-value* | *p* |
| (Intercept) | 1.93 | 1.40 – 2.46 | 7.520 | **<0.001** |
| Sampling year | 0.06 | 0.03 – 0.08 | 4.042 | **<0.001** |
| Observations | 26 | | | |
| Degrees of freedom | 24 | | | |
| R^2^ / R^2^ adjusted | 0.405 / 0.380 | | | |

Table S13. Relative density of Eurasian perch in Skrukkebukta dependent on water-temperature (Weighted-Moving-Average (WMA) over the last two years) (°C). The response is on a natural logarithmic scale.

|  | **Ln CPUE Perch (100m^2^/12h)^-1^** | | | |
| --- | --- | --- | --- | --- |
| *Predictors* | *Estimates* | *CI* | *t-value* | *p* |
| (Intercept) | -6.67 | -11.63 – -1.72 | -2.808 | **0.011** |
| Water-temperature (°C) (WMA) | 1.83 | 0.82 – 2.83 | 3.788 | **<0.001** |
| Observations | 22 | | | |
| Degrees of freedom | 20 | | | |
| R^2^ / R^2^ adjusted | 0.418 / 0.389 | | | |

Table S14. Relative density of Eurasian perch in Vaggatem dependent on water-temperature (Weighted-Moving-Average (WMA) over the last two years) (°C). The response is on a natural logarithmic scale.

|  | **Ln CPUE Perch (100m^2^/12h)^-1^** | | | |
| --- | --- | --- | --- | --- |
| *Predictors* | *Estimates* | *CI* | *t-value* | *p* |
| (Intercept) | -1.32 | -5.63 – 2.99 | -2.808 | 0.530 |
| Water-temperature (°C) (WMA) | 0.89 | 0.01 – 1.76 | 3.788 | **0.048** |
| Observations | 22 | | | |
| Degrees of freedom | 20 | | | |
| R^2^ / R^2^ adjusted | 0.182 / 0.141 | | | |

**Appendix 6: Recruitment**

Table S15. Relative proportion of one-year-old individuals in Lake Skrukkebukta and Lake Vaggatem perch populations dependent on the preceding year mean summer-water-temperature (°C, Jun-Aug) (µ=12.06, σ=1.05) in a generalized linear model with a binomial family.

|  | **Proportion of 1 year old individuals in the perch populations** | | |
| --- | --- | --- | --- |
| *Predictors* | *Estimates* | *Std. Error* | *p* |
| (Intercept) | -2.56 | 0.337 | **<0.001** |
| Preceding year, mean summer-water-temperature (°C, Jun-Aug) (lag-SWT) | 0.55 | 0.249 | **0.037** |
| Observations | 23 | | |
| Degrees of freedom | 21 | | |
| McFadden’s R^2^ | 0.205 | | |

Table S16. Relative density of one-year-old individuals in Lake Skrukkebukta and Lake Vaggatem perch populations on a natural logarithmic scale dependent on the preceding year mean summer-water-temperature (°C, Jun-Aug) (µ=12.06, σ=1.05) in a linear model.

|  | **Relative density of 1 year old individuals in the perch populations** | | |
| --- | --- | --- | --- |
| *Predictors* | *Estimates* | *CI* | *p* |
| (Intercept) | 2.25 | 1.47 – 3.03 | **<0.001** |
| Preceding year, mean summer-water-temperature (°C, Jun-Aug) (lag-SWT) | 1.18 | 0.49 – 1.87 | **0.002** |
| Observations | 23 | | |
| Degrees of freedom | 21 | | |
| R^2^ / R^2^ adjusted | 0.375 / 0.345 | | |

**Appendix 7: Back-calculated length at age**

Table S17. The relationship between back-calculated length increment (mm·year^-1^) from age 1-4 year old perch for individual cohorts and the mean summer water temperature (°C, three-year moving-average, 3YA) (µ=12.24, σ=0.49) and the mean relative density (CPUE, three-year moving average, 3YA) (µ=17.51, σ=9.9) in Lake Vaggatem and Lake Skrukkebukta (Centred and scaled predictor variables).

|  | **Length increment (mm·year^-1^) (age 1-4 years)** | | |
| --- | --- | --- | --- |
| *Predictors* | *Estimates* | *CI* | *p* |
| (Intercept) | 62.83 | 58.02 – 67.64 | **<0.001** |
| Mean relative density (CPUE, 3YA) | -6.80 | -10.44 – -3.16 | **0.001** |
| Mean summer water temperature (°C, 3YA) | 4.08 | 0.73 – 7.44 | **0.019** |
| Lake [Vaggatem] | 7.58 | 0.77 – 14.39 | **0.030** |
| Observations | 35 | | |
| R^2^ / R^2^adjusted | 0.347 / 0.283 | | |

Table S18. Back-calculated length increment for age 1 year old perch (mm·year^-1^) in Lake Vaggatem dependent on summer water temperature (°C, Jun-Aug) (µ=12.72, σ=0.98) and relative density of perch (CPUE) (µ=23.2, σ=12.22) (centred and scaled predictor variables) in a linear mixed-effect model with sample year and age when caught as random effect.

|  | **Length increment (mm·year^-1^) in age 1 year old perch** | | |
| --- | --- | --- | --- |
| *Predictors* | *Estimates* | *CI* | *p* |
| (Intercept) | 69.51 | 67.27 – 71.75 | **<0.001** |
| Summer water temperature (°C) | 0.57 | -1.72 – 2.85 | 0.618 |
| Relative density of perch (CPUE) | -0.15 | -2.27 – 1.97 | 0.887 |
| **Random Effects** | | | |
| N _year_ | 18 | | |
| N _Age_ | 5 | | |
| Observations | 407 | | |
| Degrees of freedom | 366 and 34 | | |
| Marginal R^2^ / Conditional R^2^ | 0.024 / 0.17 | | |

Table S19. Back-calculated length increment for age 1 year old perch (mm·year^-1^) in Lake Skrukkebukta dependent on summer water temperature (°C, Jun-Aug) (µ=12.31, σ=0.69) and relative density (CPUE) (µ=20.52, σ=18.49) (centred and scaled predictor variables) in a linear mixed-effect model with sample year and age when caught as random effect.

|  | **Length increment (mm·year^-1^) in age 1 year old perch** | | |
| --- | --- | --- | --- |
| *Predictors* | *Estimates* | *CI* | *p* |
| (Intercept) | 71.78 | 68.62 – 74.95 | **<0.001** |
| Summer water temperature (°C) | -0.35 | -3.20 – 2.50 | 0.803 |
| Relative density of perch (CPUE) | 0.15 | -3.37 – 3.66 | 0.932 |
| **Random Effects** | | | |
| N _year_ | 16 | | |
| N _Age_ | 5 | | |
| Observations | 292 | | |
| Degrees of freedom | 259 and 26 | | |
| Marginal R^2^ / Conditional R^2^ | 0.07 / 0.311 | | |


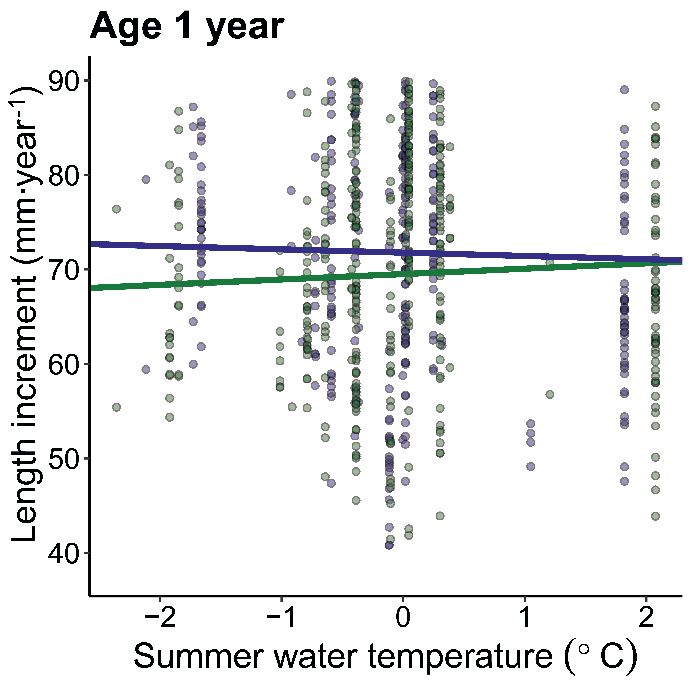

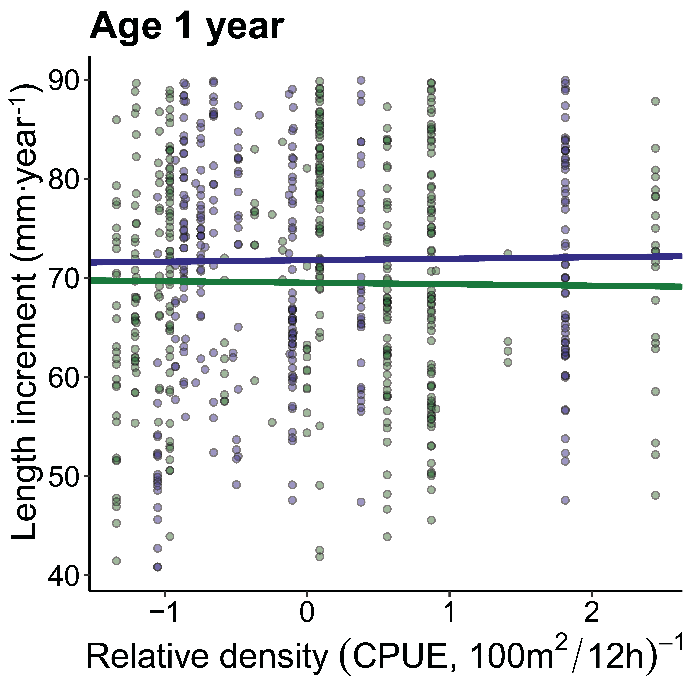


Figure S7. Back-calculated length increment for age 1 year old perch (mm·year^-1^) in Lake Skrukkebukta (blue dots, n=292) and Lake Vaggatem (green dots, n=407) dependent on summer water temperature (°C, Jun-Aug) (right) and relative density of perch (CPUE) (left) (centred and scaled). The lines describe the linear mixed-effect model results.

Length increment (mm·year^-1^) for 2 year old perch increased by 2.21 mm·°C^-1^ (Lmme: CI=0.19-3.99, p<0.001, mar-R=0.117) and 3.08 mm (Lmme: CI=1.6-4.58, p<0.001, mar-R=0.085) per degree centigrade of water temperature increment in Lake Vaggatem and Lake Skrukkebukta, respectively (Appendix Fig. S8, Table S20 & S21). For 2 year old perch, length increment decreased by 1.5 mm·10-CPUE^-1^ with relative density (CI=-2.89- -0.04, p<0.001, mar-R=0.117) in Lake Vaggatem, whereas no significant change was apparent with relative density in Lake Skrukkebukta (Appendix Fig. S8, Table S20 & S21).

Table S20. Back-calculated length increment for age 2 year old perch (mm·year^-1^) in Lake Vaggatem dependent on summer water temperature (°C, Jun-Aug) (µ=12.42, σ=0.796) and relative density of perch (CPUE) (µ=22.83, σ=15.06) (centred and scaled predictor variables) in a linear mixed-effect model with sample year and age when caught as random effect.

|  | **Length increment (mm·year^-1^) in age 2 year old perch** | | |
| --- | --- | --- | --- |
| *Predictors* | *Estimates* | *CI* | *p* |
| (Intercept) | 28.53 | 26.81 – 30.25 | **<0.001** |
| Summer water temperature (°C) | 1.76 | 0.15 – 3.18 | **<0.001** |
| Relative density of perch (CPUE) | -2.24 | -4.35 – -0.06 | **<0.001** |
| **Random Effects** | | | |
| N_Year_ | 20 | | |
| N_Age_ | 13 | | |
| Observations | 877 | | |
| Degrees of freedom | 791 and 71 | | |
| Marginal R^2^ / Conditional R^2^ | 0.117 / 0.264 | | |
|  |  | | |

Table S21. Back-calculated length increment for age 2 year old perch (mm·year^-1^) in Lake Skrukkebukta dependent on summer water temperature (°C, Jun-Aug) (µ=12.50, σ=0.84) and relative density (CPUE) (µ=17.7, σ=13.54) (centred and scaled predictor variables) in a linear mixed-effect model with sample year and age when caught as random effect.

|  | **Length increment (mm·year^-1^) in age 2 year old perch** | | |
| --- | --- | --- | --- |
| *Predictors* | *Estimates* | *CI* | *p* |
| (Intercept) | 29.22 | 26.74 – 31.70 | **<0.001** |
| Summer water temperature (°C) | 2.59 | 1.34 – 3.85 | **<0.001** |
| Relative density of perch (CPUE) | -1.00 | -2.74 – 0.74 | 0.252 |
| **Random Effects** | | | |
| N_Year_ | 10 | | |
| N_Age_ | 12 | | |
| Observations | 580 | | |
| Degrees of freedom | 515 and 51 | | |
| Marginal R^2^ / Conditional R^2^ | 0.085 / 0.278 | | |


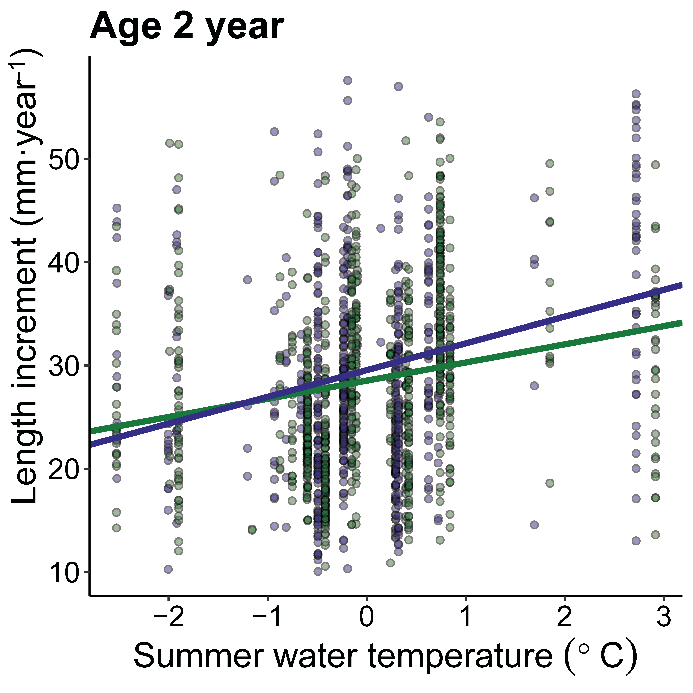

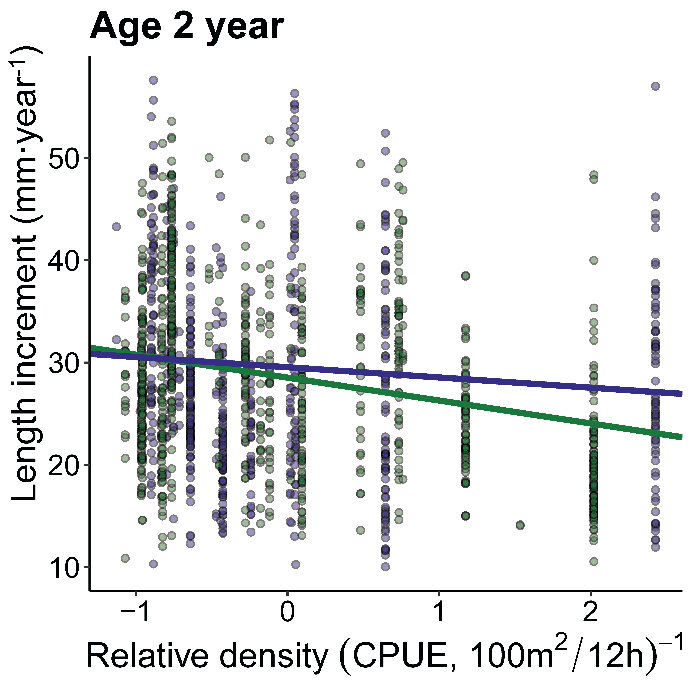


Figure S8. Back-calculated length increment for age 2-year-old perch (mm·year^-1^) in Lake Skrukkebukta (blue dots, n=580) and Lake Vaggatem (green dots, n=877) dependent on summer water temperature (°C, Jun-Aug) (right) and relative density of perch (CPUE) (left) (centred and scaled). The lines describe the linear mixed-effect model results.

Length increment (mm·year^-1^) for 3-year-old perch increased by 1.59 mm·°C^-1^ (Lmme: CI=0.21-2.98, p=0.025, mar-R=0.07) and 1.21 mm·°C^-1^ (Lmme: CI=0.24-2.2, p=0.031, mar-R=0.022) in water temperature increase in Lake Vaggatem and Lake Skrukkebukta, respectively (Appendix Fig. S9, Table S22 & S23). For 3-year-old perch, length increment decreased by 1.1 mm per 10-CPUE increment in relative density (CI=-1.93- -0.16, p=0.021, mar-R=0.07) in Lake Vaggatem, whereas no significant change was apparent with relative density in Lake Skrukkebukta (Appendix Fig. S9, Table S22 & S23).

Table S22. Back-calculated length increment for age 3-year-old perch (mm·year^-1^) in Lake Vaggatem dependent on summer water temperature (°C, Jun-Aug) (µ=12.31, σ=0.82) and relative density of perch (CPUE) (µ=20.24, σ=14.72) (centred and scaled predictor variables) in a linear mixed-effect model with sample year and age when caught as random effect.

|  | **Length increment (mm·year^-1^) in age 3 year old perch** | | |
| --- | --- | --- | --- |
| *Predictors* | *Estimates* | *CI* | *p* |
| (Intercept) | 26.04 | 24.88 – 27.20 | **<0.001** |
| Summer water temperature (°C) | 1.30 | 0.17 – 2.44 | **0.025** |
| Relative density of perch (CPUE) | -1.55 | -2.85 – -0.24 | **0.021** |
| **Random Effects** | | | |
| N_Year_ | 11 | | |
| N _Age_ | 11 | | |
| Observations | 791 | | |
| Degrees of freedom | 719 and 59 | | |
| Marginal R^2^ / Conditional R^2^ | 0.069 / 0.363 | | |

Table S23. Back-calculated length increment for age 3-year-old perch (mm·year^-1^) in Lake Skrukkebukta dependent on summer water temperature (°C, Jun-Aug) (µ=12.15, σ=0.85) and relative density of perch (CPUE) (µ=16.05, σ=9.99) (centred and scaled predictor variables) in a linear mixed-effect model with sample year and age when caught as random effect.

|  | **Length increment (mm·year^-1^) in age 3 year old perch** | | |
| --- | --- | --- | --- |
| *Predictors* | *Estimates* | *CI* | *p* |
| (Intercept) | 20.35 | 19.31 – 21.40 | **<0.001** |
| Summer water temperature (°C) | 1.03 | 0.2 – 1.88 | **0.031** |
| Relative density of perch (CPUE) | -0.07 | -0.99 – 0.93 | 0.883 |
| **Random Effects** | | | |
| N _Year_ | 10 | | |
| N _Age_ | 10 | | |
| Observations | 503 | | |
| Degrees of freedom | 439 and 47 | | |
| Marginal R^2^ / Conditional R^2^ | 0.022/0.193 | | |


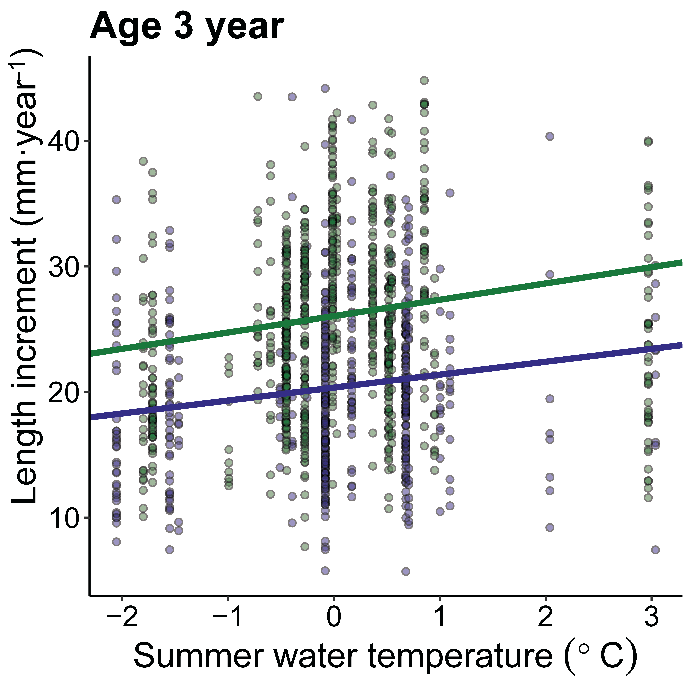

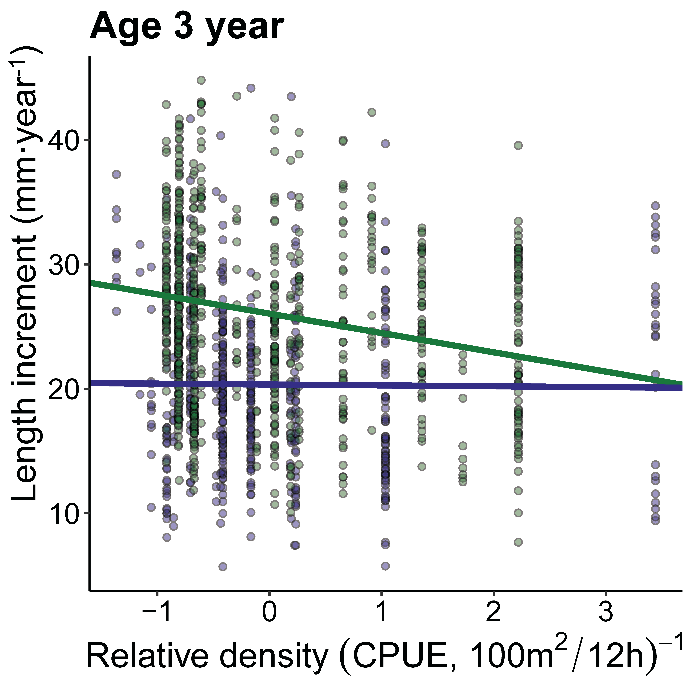


Figure S9. Back-calculated length increment for age 3 year old perch (mm·year^-1^) in Lake Skrukkebukta (blue dots, n=503) and Lake Vaggatem (green dots, n=791) dependent on summer water temperature (°C, Jun-Aug) (right) and relative density of perch (CPUE) (left) (centred and scaled). The lines describe the linear mixed-effect model results.

Length increment (mm·year^-1^) for 4 year old perch increased by 2.24 mm (Lmme: CI=1.03-3.21, p<0.001, mar-R=0.09) and 1.82 mm (Lmme: CI=0.65-3.51, p=0.005, mar-R=0.097) per degree centigrade increment in water temperature in Lake Vaggatem and Lake Skrukkebukta, respectively (Appendix Fig. S10, Table S24 & S25). For 4 year old perch, length increment did not significantly change with relative density (Appendix Fig. S10, Table S24 & S25).

Table S24. Back-calculated length increment for age 4 year old perch (mm·year^-1^) in Lake Vaggatem dependent on summer water temperature (°C, Jun-Aug) (µ=12.31, σ=0.98) and relative density of perch (CPUE) (µ=23.6, σ=12.25) (centred and scaled predictor variables) in a linear mixed-effect model with sample year and age when caught as random effect.

|  | **Length increment (mm·year^-1^) in age 4 year old perch** | | |
| --- | --- | --- | --- |
| *Predictors* | *Estimates* | *CI* | *p* |
| (Intercept) | 22.11 | 20.99 – 23.23 | **<0.001** |
| Summer water temperature (°C) | 2.17 | 1.05 – 3.28 | **<0.001** |
| Relative density of perch (CPUE) | -0.57 | -1.67 – 0.53 | 0.301 |
| **Random Effects** | | | |
| N _Year_ | 11 | | |
| N _Age_ | 11 | | |
| Observations | 761 | | |
| Degrees of freedom | 689 and 59 | | |
| Marginal R^2^ / Conditional R^2^ | 0.09 / 0.404 | | |

Table S25. Back-calculated length increment for age 4-year-old perch (mm·year^-1^) in Lake Skrukkebukta dependent on summer water temperature (°C, Jun-Aug) (µ=11.87, σ=1.07) and relative density of perch (CPUE) (µ=14.26, σ=7.16) (centred and scaled predictor variables) in a linear mixed-effect model with sample year and age when caught as random effect.

|  | **Length increment (mm·year^-1^) in age 4 year old perch** | | |
| --- | --- | --- | --- |
| *Predictors* | *Estimates* | *CI* | *p* |
| (Intercept) | 18.11 | 16.67 – 19.56 | **<0.001** |
| Summer water temperature (°C) | 1.95 | 0.61 – 3.28 | **0.005** |
| Relative density of perch (CPUE) | -0.19 | -1.33 – 0.96 | 0.745 |
| **Random Effects** | | | |
| N _Year_ | 10 | | |
| N _Age_ | 10 | | |
| Observations | 474 | | |
| Degrees of freedom | 416 and 46 | | |
| Marginal R^2^ / Conditional R^2^ | 0.097 / 0.296 | | |
|  |  | | |
|  |  |  |  |


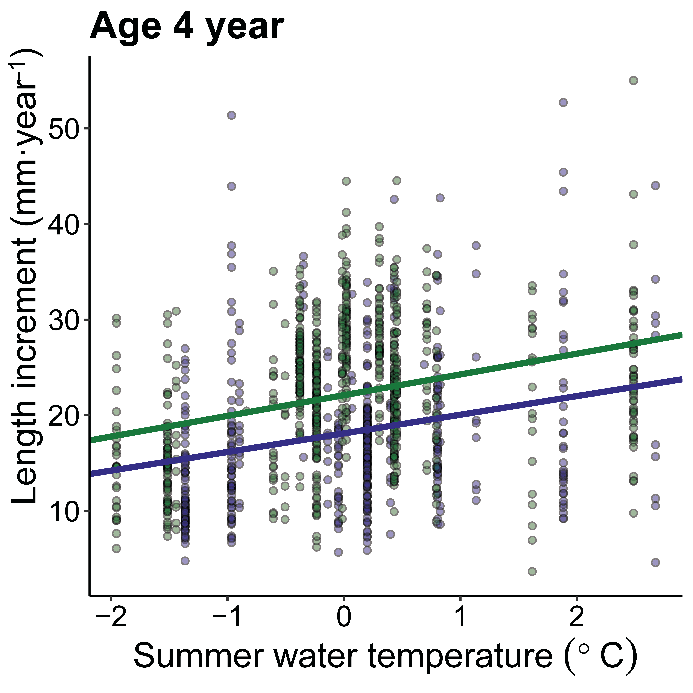

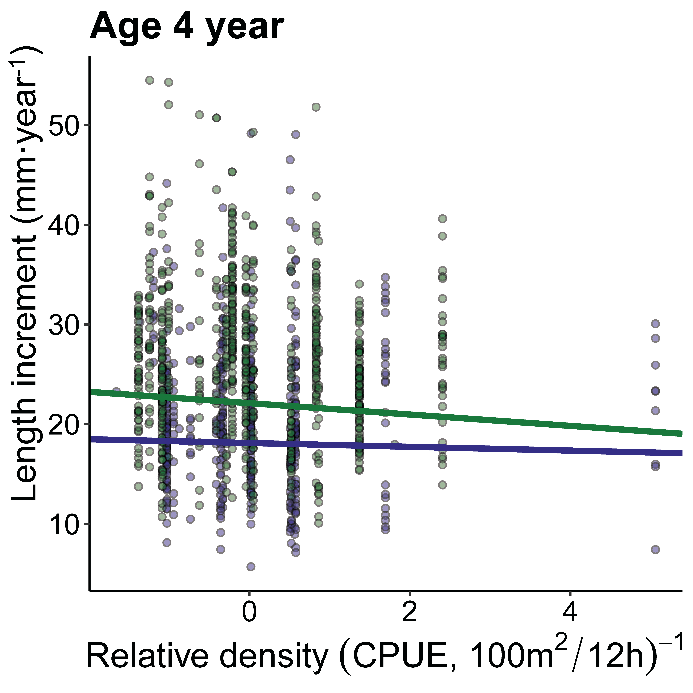


Figure S10. Back-calculated length increment for age 4 year old perch (mm·year^-1^) in Lake Skrukkebukta (blue dots, n=474) and Lake Vaggatem (green dots, n=767) dependent on summer water temperature (°C, Jun-Aug) (right) and relative density of perch (CPUE) (left) (centred and scaled). The lines describe the linear mixed-effect model results.

**Appendix 8: Age at maturation and sex ratio**


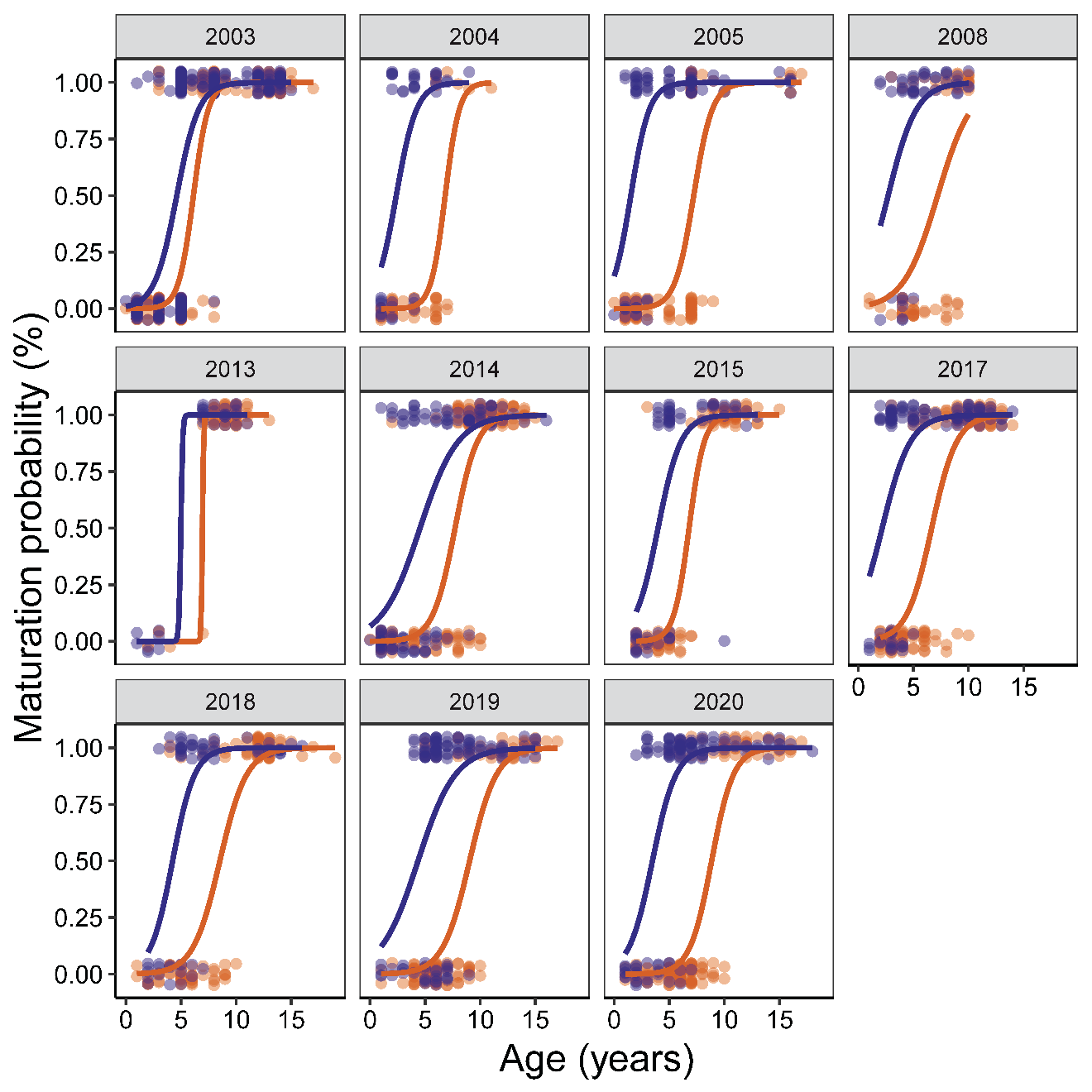


Figure S11. Probability of being mature dependent on age of the perch with logistic regression lines for the individual sampled cohorts. Blue dots and lines depict male individuals. Orange dots and lines depict female individuals.

Cohort-specific age at maturation was not dependent on the difference in sex ratio (proportion of females) for the different cohorts within the perch populations (fig. S12) (p=0.92, t=0.102 on 15 d.f., adj-R^2^=-0.07). The sex ratio (proportion of females) was between 0.48-0.72 for the different cohorts, with no change in sex ratio over time (p=0.21, t=-1.31 on 15 d.f., adj-R^2^=0.04).


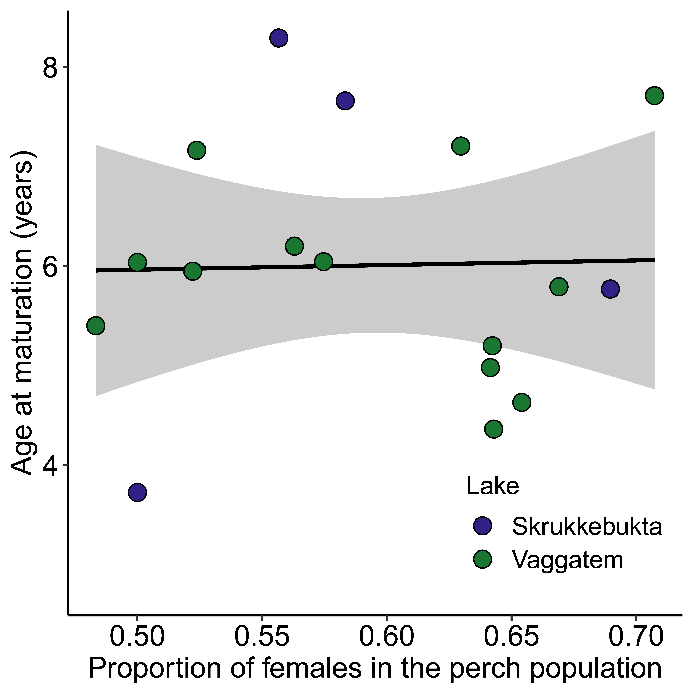


Figure S12. Shows how the difference in proportion of females in the different cohorts affects the age at maturation estimated for the different cohorts (n=16). The black line depicts results from the linear model with the shaded area describes the 95% confidence interval.

Table S26. Cohort-specific age at maturation (A_50_, from logistic regression, fig. S11) dependent on back-calculated length increment (mm·year^-1^) from age 1-4 years.

|  | **A_50_** | | |
| --- | --- | --- | --- |
| *Predictors* | *Estimates* | *CI* | *p* |
| (Intercept) | 11.58 | 8.43 – 14.74 | **<0.001** |
| Length increment (1-4 years) | -0.08 | -0.12 – -0.03 | **0.002** |
| Observations | 16 | | |
| R^2^ / R^2^adjusted | 0.506 / 0.47 | | |

**
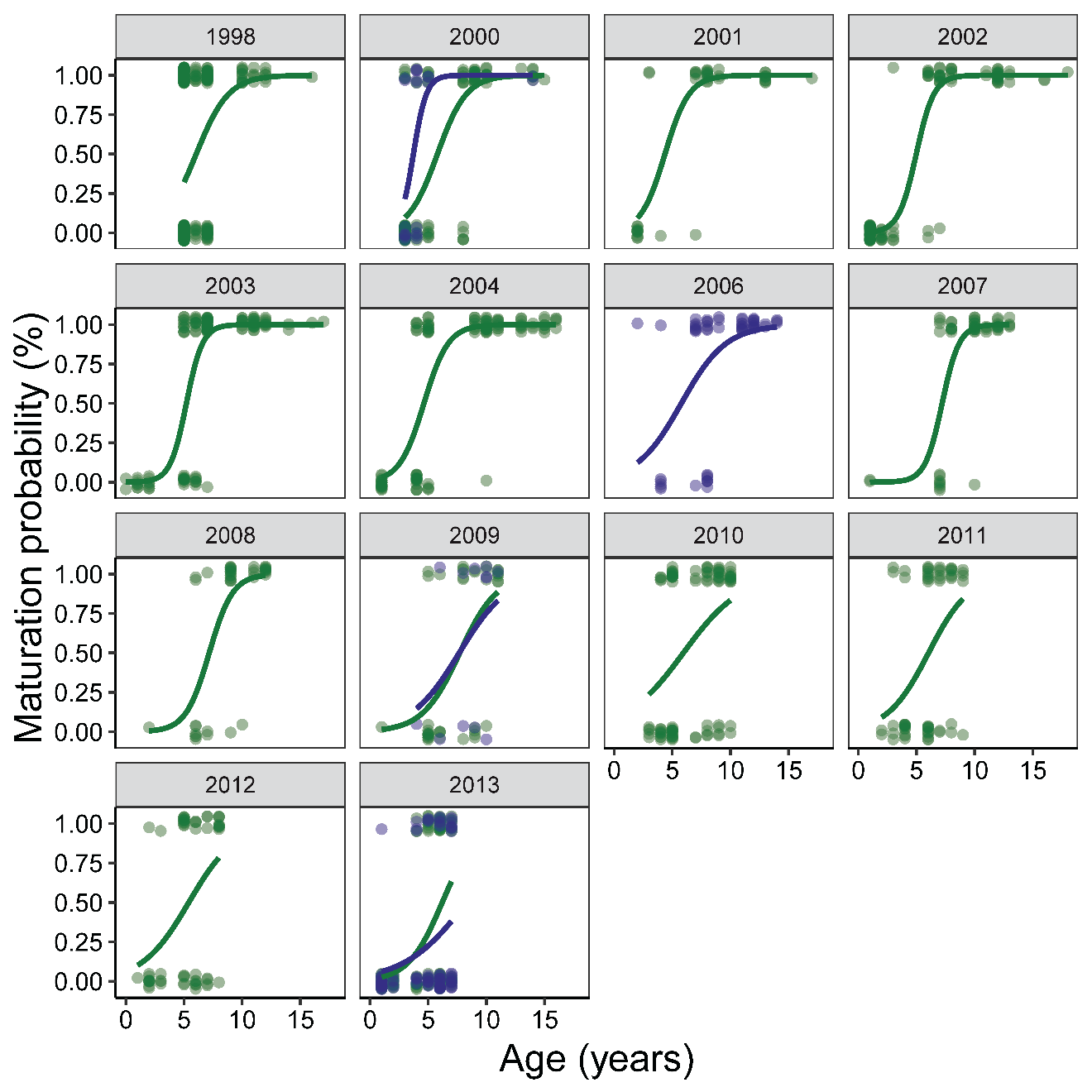
**

Figure S13. Probability of being mature dependent on age of the perch with logistic regression lines for the individual sampled cohorts. Blue dots and lines depict individuals sampled in Lake Skrukkebukta. Green dots and lines depict individuals sampled in Lake Vaggatem.

**Appendix 9: Relative density of whitefish**


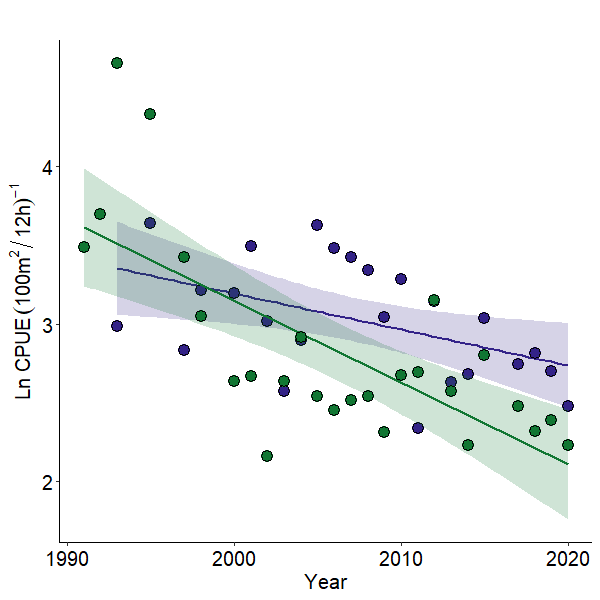


Figure S14. Relative density (ln-CPUE, no. of fish per 100m^2^ per 12h) of European whitefish in the littoral zone of Lake Vaggatem (green, n=26) and Lake Skrukkebukta (blue, n=24) from year 1990 to year 2020.

Table S27. Summary results for the linear model of the development relative density (CPUE) of whitefish (*Coregonus lavaretus*) in the littoral zone of Lake Vaggatem over the 30-year sampling period. The response is on a natural logarithmic scale.

|  | **Ln CPUE Whitefish (100m^2^/12h)^-1^** | | | |
| --- | --- | --- | --- | --- |
| *Predictors* | *Estimates* | *CI* | *t-value* | *p* |
| (Intercept) | 3.61 | 3.21 – 4.02 | 18.3 | **<0.001** |
| Sampling year | -0.05 | -0.07 – -0.03 | -4.91 | **<0.001** |
| Observations | 26 | | | |
| Degrees of freedom | 24 | | | |
| R^2^ / R^2^ adjusted | 0.501/ 0.479 | | | |

Table S28. Summary results for the linear model of the development relative density (CPUE) of whitefish (*Coregonus lavaretus*) in the littoral zone of Lake Skrukkebukta over the 30-year sampling period. The response is on a natural logarithmic scale.

|  | **Ln CPUE Whitefish (100m^2^/12h)^-1^** | | | |
| --- | --- | --- | --- | --- |
| *Predictors* | *Estimates* | *CI* | *t-value* | *p* |
| (Intercept) | 3.39 | 3.03 – 3.75 | 19.32 | **<0.001** |
| Sampling year | -0.02 | -0.04 – -0.01 | -2.60 | **=0.016** |
| Observations | 24 | | | |
| Degrees of freedom | 22 | | | |
| R^2^ / R^2^ adjusted | 0.235/ 0.20 | | | |

**References**

Alabaster, J. S. & Downing, A. L. 1966. A field and laboratory investigation of the effect of heated effluents on fish. *Fisheries Investigation Series, London, 6.*

Amundsen, P.-A., Bøhn, T., Popova, O. A., Staldvik, F. J., Reshetnikov, Y. S., Kashulin, N. A., & Lukin, A. A. 2003. Ontogenetic niche shifts and resource partitioning in a subarctic piscivore fish guild. *Hydrobiologia*, *497*, 109-119.

Craig, J. F. 1978. A study of the food and feeding of perch, *Perca fluviatilis* L., in Windemere. *Freshwater Biology 8,* 59-68.

Dahlke, F. T., Wohlrab, S., Butzin, M., & Pörtner, H. O. 2020. Thermal bottlenecks in the life cycle define climate vulnerability of fish. *Science*, *369,* 65–70. <https://doi.org/10.1126/science.aaz3658>

FAO. 2020. Species fact sheet: *Perca fluivitalis*. *Food and Agriculture Organization of the United Nations*. http://www.fao.org/fishery/species/2298/en

Hayden, B., Harrod, C., & Kahilainen, K. K. 2014. Lake morphometry and resource polymorphism determine niche segregation between cool- and cold-water-adapted fish. *Ecology, 95,* 538–552. https://doi.org/10.1890/13-0264.1

Hjelm, J., Svanbäck, R., Byström, P., Persson, L., & Wahlström, E. 2001. Diet-dependent body morphology and ontogenetic reaction norms in Eurasian perch. *Oikos, 95,* 311–323. https://doi.org/10.1034/j.1600-0706.2001.950213.x

Hoestlandt, H., & Devienne, A. 1980. A more precise statement of the relation between incubation time and temperature in the teleosteen fish, *Perca fluviatilis* L. *Comptes Rendus Hebdomadaires des Seances de l'Academie des Sciences. Serie D (France), 290*, 1123-1125.

Hokanson, K. E. F., & Kleiner, C. F. 1974. Effects of constant and rising temperatures on survival and developmental rates of embryonic and larval yellow perch, *Perca flavescens* (Mitchill). In *The early life history of fish,* pp. 437-448. Springer, Berlin, Heidelberg.

Hokanson, K. E. F. 1977. Temperature requirements of some percids and adaptations to the seasonal temperature cycle. *Journal of the Fisheries Research Board of Canada, 34,* 1524–1550.

Horoszewicz, L. 1973. Lethal temperatures of roach fry (*Rutilus rutilus* L.) from lakes with normal and artificially elevated temperature. *Polskie Archiwum Hydrobiologii, 18,* 69-79.

Karås, P. 1987. Food consumption, growth and recruitment in perch (*Perca fluviatilis* L.). Ph.D. thesis, Uppsala University, Uppsala, Sweden. pp. 129.

Karås, P. 1990. Seasonal changes in growth and standard metabolic rate of juvenile perch, *Perca fluviatilis* L. *Journal of Fish Biology, 37,* 913– 920.

Karås, P., & Thoresson, G. 1992. An application of a bioenergetics model to Eurasian perch (*Perca fluviatilis* L.). *Journal of Fish Biology, 41,* 217-230.

Karås, P. 1996. Basic abiotic conditions for production of perch (*Perca fluviatilis* L.) young- of-the-year in the Gulf of Bothnia. *Annales Zoologici Fennici, 33,* 371-381.

Küttel, S., Peter, A., & Wüest A. 2002. Temperaturpräferenzen und-limiten von Fischarten Schweizerischer Fliessgewässer. *Rhône Revitalisierung, 1,* pp. 41*.*

Mooij, W. M., Van Rooij, J. M., & Wijnhoven, S. 1999. Analysis and comparison of fish growth from small samples of length-at-age data: Detection of sexual dimorphism in Eurasian perch as an example. *Transactions of the American Fisheries Society*, *128,* 483–490. https://doi.org/10.1577/1548-8659(1999)128<0483:aacofg>2.0.co;2

Olin, M., Jutila, J., Lehtonen, H., Vinni, M., Ruuhijärvi, J., Estlander, S., Rask, M., Kuparinen, A., & Lappalainen, J. 2012. Importance of maternal size on the reproductive success of perch, *Perca fluviatilis*, in small forest lakes: implications for fisheries management. *Fisheries Management and Ecology, 19,* 363–374. https://doi.org/10.1111/j.1365-2400.2012.00845.x

Persson, L. 1988. Asymmetries in competitive and predatory interactions in fish populations. *Size-structured populations,* pp. 203-218. Springer, Berlin, Heidelberg.

Saat, T. A., Saat, V., & Veersalu, Т. 1996. The rate of early development in perch *Perca fluviatilis* L. and ruffe *Gymnocephalus cernuus* (L.) at different temperatures. *Annales Zoologici Fennici, 33,* 693-698.

Sandlund, O. T., Gjelland, K. Ø., Bøhn, T., Knudsen, R., & Amundsen, P.-A. 2013. Contrasting population and life history responses of a young morph-pair of European whitefish to the invasion of a specialised coregonid competitor, vendace. *PloS One, 8,* e68156. https://doi.org/ARTN e68156 10.1371/journal.pone.0068156

Shuter, B. J., Finstad, A. G., Helland, I. P., Zweimüller, I., & Hölker, F. 2012. The role of winter phenology in shaping the ecology of freshwater fish and their sensitivities to climate change. *Aquatic Sciences*, *74,* 637-657*.*

Willemsen, J., 1977. Population dynamics of percids in Lake Issel and some smaller lakes in the Netherlands. *Journal of the Fisheries Research Board of Canada, 34,* 1710–1719.
